# Stratification and prognostic evaluation of breast cancer subtypes defined by obesity-associated genes

**DOI:** 10.1007/s12672-024-00988-0

**Published:** 2024-04-27

**Authors:** Dongjuan Chen, Zilu Xie, Jun Yang, Ting Zhang, Qiliang Xiong, Chen Yi, Shaofeng Jiang

**Affiliations:** 1 Department of Laboratory Medicine, Maternal and Child Health Hospital of Hubei Province, Tongji Medical College, Huazhong University of Science and Technology, Wuhan, 430070 China; 2 Department of Biomedical Engineering, Nanchang Hang Kong University, Jiangxi, 330063 China

**Keywords:** Obesity, Inflammation, Subtype identification, Diagnosis

## Abstract

**Objective:**

Breast cancer was the most common type of cancer among women worldwide, significantly impacting their quality of life and survival rates. And obesity has been widely accepted as an important risk factor for breast cancer. However, the specific mechanisms by which obesity affects breast cancer were still unclear. Therefore, studying the impact mechanisms of obesity as a risk factor for breast cancer was of utmost importance.

**Methods:**

This study was based on TCGA breast cancer RNA transcriptomic data and the GeneCard obesity gene set. Through single and multiple factor Cox analysis and LASSO coefficient screening, seven hub genes were identified. The independent mechanisms of these seven hub genes were evaluated from various aspects, including survival data, genetic mutation data, single-cell sequencing data, and immune cell data. Additionally, the risk prognosis model and the neural network diagnostic model were established to further investigate these seven hub genes. In order to achieve precision treatment for breast cancer (BRCA), based on the RNA transcriptomic data of the seven genes, 1226 BRCA patients were divided into two subtypes: BRCA subtype 1 and BRCA subtype 2. By studying and comparing the immune microenvironment, investigating the mechanisms of differential gene expression, and exploring the mechanisms of subnetworks, we aim to explore the clinical differences in the presentation of BRCA subtypes and achieve precision treatment for BRCA. Finally, qRT-PCR experiments were conducted to validate the conclusions of the bioinformatics analysis.

**Results:**

The 7 hub genes showed good diagnostic independence and can serve as excellent biomarkers for molecular diagnosis. However, they do not perform well as independent prognostic molecular markers for BRCA patients. When predicting the survival of BRCA patients, their AUC values at 1 year, 3 years, and 5 years are mostly below 0.5. Nevertheless, through the establishment of the risk prognosis model considering the combined effect of the seven hub genes, it was found that the survival prediction of BRCA patients can be significantly improved. The risk prognosis model, compared to the independent use of the seven hub genes as prognostic markers, achieved higher timeROC AUC values at 1 year, 3 years, and 5 years, with values of 0.651, 0.669, and 0.641 respectively. Additionally, the neural network diagnostic model constructed from the 7 genes performs well in diagnosing BRCA, with an AUC value of 0.94, accurately identifying BRCA patients. The two subtypes identified by the seven hub genes exhibited significant differences in survival period, with subtype 1 having a poor prognosis. The differential mechanisms between the two subtypes mainly originate from regulatory differences in the immune microenvironment. Finally, the results of this study’s bioinformatics analysis were validated through qRT-PCR experiments.

**Conclusion:**

7 hub genes serve as excellent independent biomarkers for molecular diagnosis, and the neural network diagnostic model can accurately distinguish BRCA patients. In addition, based on the expression levels of these seven genes in BRCA patients, two subtypes can be reliably identified: BRCA subtype 1 and BRCA subtype 2, and these two subtypes showed significant differences in BRCA patient survival prognosis, proportion of immune cells, and expression levels of immune cells. Among them, patients with subtype 1 of BRCA had a poor prognosis.

**Supplementary Information:**

The online version contains supplementary material available at 10.1007/s12672-024-00988-0.

## Introduction

Breast cancer was the most common type of cancer among women worldwide, significantly impacting their quality of life and survival rates [1–3]. According to global cancer statistics, the incidence and mortality rates of breast cancer have been continuously increasing in recent years, highlighting the importance of in-depth research on risk factors and pathogenesis of breast cancer [4–6]. The pathogenesis of breast cancer was extremely complex, involving genetic factors, age, hormone levels, lifestyle, and other factors [7–9]. In recent years, obesity had gained attention as a potential risk factor [10], particularly with the rapid increase in obesity rates globally and the associated health issues, including the risk of breast cancer [11–13]. However, the direct impact of obesity on the incidence and severity of breast cancer, as well as the underlying biological mechanisms, remains unclear [14]. A deeper understanding of these issues will provide new insights into the etiology of breast cancer and contribute to the development of new strategies for prevention and treatment.

Obesity has been widely accepted as an important risk factor for breast cancer [15–17]. As early as 2018, Nattenmuller et al. found through histopathological analysis of 657 breast cancer patients that obesity was associated with a lower risk of invasive breast tumors [18]. Subsequently, in 2019, it was discovered that obesity may influence the hormone receptor-positive breast cancer-specific mortality by promoting unfavorable tumor prognosis [19]. However, the specific biological mechanisms by which obesity contributes to breast cancer are not yet clear [20]. Some studies suggested that the correlation between obesity and breast cancer may be attributed to systemic and local changes caused by obesity, such as elevated levels of insulin, glucose, and hormones derived from adipose tissue. These factors can regulate metabolic pathways in breast cancer cells and the breast microenvironment, leading to metabolic dysregulation and increased risk of breast cancer [21]. Other research suggested that the correlation between obesity and breast cancer may involve cross-communication between adipocytes and cancer cells in the tumor microenvironment. Key players in this process include adipocyte-derived adiponectin transferase and cancer cell-derived acyl-CoA synthetase ACSBG1, which play critical roles in obesity-driven breast cancer progression [22]. Additionally, obesity may alter the tumor microenvironment of breast cancer by changing the composition and function of adipocytes, stromal cells, immune cells, endothelial cells, and extracellular matrix. These changes enhanced the secretion of cytokines and adipokines, as well as the local levels of estrogen in the breast tumor microenvironment, promoting rapid tumor growth and resistance to treatment [23]. However, despite the progress made in understanding the relationship between obesity and breast cancer risk, the complex and heterogeneous mechanisms underlying breast cancer pathogenesis, particularly at the level of gene expression and transcriptomics, remain unclear and require further investigation. Additionally, the discovery of obesity-related biomarkers and their specific diagnostic and prognostic value in relation to breast cancer also requires more in-depth research and validation. In recent studies, there is evidence indicating that obesity in patients may play a significant role in the heterogeneity of breast cancer and should be considered for personalized adjustments in breast cancer treatment [24]. Therefore, the concurrent emergence of different breast cancer subtypes potentially induced by obesity is another focal point that decidedly deserves attention. And this will hold significant implications for the advancement of precision individualized treatment, particularly in BRCA patients.

**Fig. 1:**
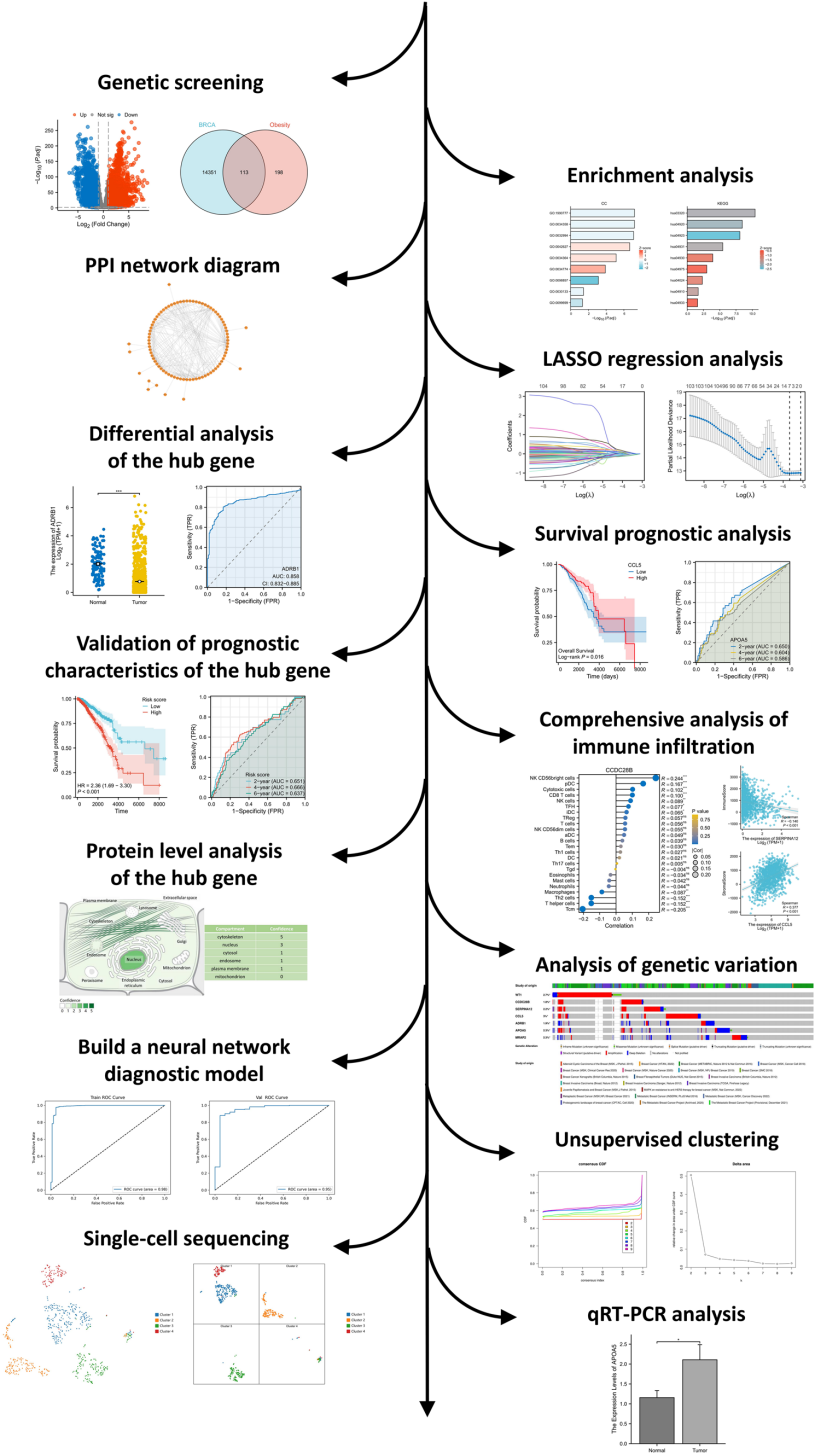
The specific process of this study

This study aimed to explore the impact of obesity on the pathogenesis of breast cancer and identified potential novel biomarkers through comprehensive bioinformatics analysis. The specific workflow was illustrated in Fig. 1, Which involves integrating transcriptomic data and single-cell sequencing data from both the TCGA and GEO databases to identify key genes associated with obesity and subtype identification. Multiple analyses will be conducted to evaluate the mechanistic effects and clinical significance of these key genes in breast cancer (BRCA). Overall, the goal of this study was to uncover how obesity influences the development of breast cancer and identify genes that may play a crucial role in this process. These findings were expected to provide new strategies for the prevention and treatment of breast cancer.

## Method

### Data acquisition

Obtained RNA-seq transcriptome data from 1226 human breast tissue samples, including 1113 breast cancer tumor samples and 113 normal samples, from The Cancer Genome Atlas (TCGA, https://portal.gdc.cancer.gov). Additionally, downloaded clinical information of breast cancer patients from the TCGA database. Retrieved 9912 obesity-related genes from GeneCards (https://www.genecards.org) and selected 311 genes with correlation coefficients greater than 7. Retrieved mutation information of 7 hub genes in protein structures from the cBioPortal database (cbioportal.org). Acquired 750 Sorted Cells from Human Invasive Ductal Carcinoma, 3′LT v3.1 dataset from the 10X Genomics database (https://www.10xgenomics.com/). Also obtained the major regulatory genes of immune cells CD8 T cells and DC from the Cell Marker database (http://xteam.xbio.top/CellMarker/) for single-cell sequencing. Finally, drug sensitivity data was obtained through the CellMiner database (https://discover.nci.nih.gov/cellminer/home.do).

### Retrieve the intersecting genes

Differential analysis was performed on breast cancer RNA-seq transcriptome data from the TCGA database using the R package “DESeq2”. A total of 11,991 differentially expressed genes were obtained. Subsequently, the intersection analysis was conducted between these 11,991 differentially expressed genes and 311 obesity-related genes, resulting in 113 intersecting genes. The criteria for differential analysis were set as |log2FoldChange|> 1 and padj < 0.05 for filtering.

### Gene mechanism research

In order to gain a more intuitive understanding of the direct physical interactions or indirect functional connections among these 113 intersecting genes, GO enrichment analysis and KEGG enrichment analysis were performed on the intersecting genes using the R package “clusterProfiler”. Additionally, the STRING database and Cytoscape software (v3.7.2) were utilized to construct the protein–protein interaction (PPI) network among the intersecting genes.

### The acquisition and preliminary evaluation of hub genes

In order to further investigate the impact of obesity on breast cancer patients, further screening was conducted on 113 intersecting genes through LASSO regression analysis. Seven hub genes with non-zero penalty factors (λ) were identified: ADRB1, APOA5, CCDC28B, CCL5, MRAP2, SERPINA12, and WT1. The aforementioned LASSO regression analysis was performed using the R package “glmnet”. Additionally, independent diagnostic models for these seven hub genes were established using the R package “pROC”. Furthermore, correlation analysis was conducted to explore the potential connections among these seven hub genes in breast cancer patients.

To further investigate the role of the proteins encoded by the 7 hub genes in BRCA, the protein levels of these genes in tumor and healthy breast tissues were analyzed using the Human Protein Atlas database (https://www.proteinatlas.org, HPA). Additionally, the protein level distribution and localization of the proteins encoded by the 7 hub genes were obtained from the GeneCards database (https://www.genecards.org).

### Independent prognostic evaluation of hub genes

To investigate whether the 7 hub genes can serve as independent prognostic factors, survival analysis was performed on each of the 7 hub genes using the R packages “survival,” “survminer,” and “ggplot2.” Additionally, to assess the accuracy of the independent survival prognostic model, time-dependent ROC curves were plotted using the R package “timeROC” based on the sample data. The survival prediction performance of the 7 genes was evaluated separately for BRCA patients at 2, 4, and 6 years.

### The establishment of the risk prognostic model

Perform Cox univariate and multivariate regression analysis on 7 hub genes using R packages “glmnet” and “survival”. Calculate the risk score based on Cox hazard coefficients and establish a risk prognostic model. The risk score was calculated according to the following formula:

$$\user2{Risk score} = {\varvec{Coef}}\left( {{\varvec{Cox}}} \right) \times {\varvec{Exp}}\left( {\varvec{k}} \right)$$

where ***Exp (k)*** represents the expression level of gene k, and ***Coef (Cox)*** represents the Cox hazard coefficient.

Additionally, to evaluate the predictive effect of the risk prognostic model on the survival time of BRCA patients at 2, 4, and 6 years, timROC curves were plotted based on the “timeROC” package.

### Analysis of the immune microenvironment

The correlation between 24 immune cells and 7 hub genes was analyzed using the R package “ssGSEA” and visualized. Additionally, the immune score and stromal score of the 7 hub genes were calculated based on the ESTIMATE algorithm.

### The construction of the neural network diagnostic model

The diagnostic model was constructed based on the feedforward neural network algorithm to investigate whether the collective interaction of 7 hub genes can effectively distinguish breast cancer patients from normal individuals. The binary cross-entropy loss function was used as the model’s loss function when constructing the diagnostic model [25]. The formula for calculating this function was:

$${\varvec{BCELoss}} = - \frac{{\mathbf{1}}}{{\varvec{N}}}\mathop \sum \limits_{{{\varvec{i}} = {\mathbf{1}}}}^{{\varvec{N}}} \left[ {{\varvec{y}}_{{\varvec{i}}} {\mathbf{log}}\left( {{\varvec{p}}_{{\varvec{i}}} } \right) + \left( {{\mathbf{1}} - {\varvec{y}}_{{\varvec{i}}} } \right){\mathbf{log}}\left( {{\mathbf{1}} - {\varvec{p}}_{{\varvec{i}}} } \right)} \right]$$

We set the learning rate of the model to 0.1 and the weight decay to 0.001. Additionally, the RNA-seq transcriptome dataset was divided into a training set and a validation set. The training set consisted of 890 BRCA samples and 90 normal samples, while the validation set consisted of 223 BRCA samples and 23 normal samples. The training set data was used to train the diagnostic model for 300 iterations, and then the validation set data was used to evaluate the diagnostic performance of the constructed model.

### The identification of BRCA subtypes

Perform unsupervised clustering on breast cancer sample data using the ConsensusClusterPlus algorithm. First, select 80% of the sample data and all gene data for clustering analysis using the R package “ConsensusClusterPlus”. Set the maximum K value for clustering as 9, random seed as 102, and repeat the training 500 times. Then, use the PAC algorithm to determine the optimal K value as 2, which divides BRCA into two subtypes.

Combine the two subtypes with clinical survival data and plot Kaplan–Meier survival curves. Additionally, to further evaluate the stability of the two subtypes, perform clustering on the two subtypes using the t-SNE algorithm. Download the dataset “LM22” containing 22 immune cell data from the CIBERSORT website (https://cibersortx.stanford.edu/). Use the R packages “e1071”, “parallel”, and “preprocessCore” to calculate the infiltration of 22 immune cell types in the two BRCA subtypes using the CIBERSORT algorithm and visualize the results. To investigate potential differences in the mechanisms of action between the two BRCA subtypes, perform differential analysis on the transcriptome of the two subtypes to obtain 1533 differentially expressed genes. Conduct enrichment analysis on these genes and construct a protein–protein interaction (PPI) network. Use the plugins “cytoHubba” and “MCODE” in Cytoscape software to select 30 hub genes and two subnetworks. Perform enrichment analysis on the 30 hub genes and two subnetworks separately.

To minimize subjective judgment in the ConsensusClusterPlus algorithm, perform clustering analysis on the sample data again using the non-negative matrix factorization (NMF) algorithm. Use the R packages “NMF”, “Rmpi”, and “doMPI”, select the brunt method, set the rank as 6, and iterate 10 times to select the optimal rank value. Calculate the confusion matrix and consensus matrix to visualize the stability of the subtype clustering.

### The identification of cell populations

Identification of cell populations in the 750 Sorted Cells from Human Invasive Ductal Carcinoma, 3′ LT v3.1 dataset was performed using the “CellRanger” software (v 7.1.0) provided by 10X for single-cell sequencing upstream analysis. The resulting cloupe file was imported into the “Loupe Browser” software (v 6.5.0) provided by 10X for t-SNE algorithm-based clustering of 699 cells, resulting in 4 cell subtypes. Marker genes for CD8 T cells and DC were obtained from the CellMarker database, and the 4 cell subgroups were re-clustered.

### qRT-PCR analysis

With the approval of Wuhan Maternal and Child Health Hospital in Hubei Province, quantitative real-time PCR experiments were conducted on serum samples from 10 healthy individuals and 11 patients with BRCA. All volunteers participating in the experiment signed informed consent forms. SYBR FAST qPCR Master Mix was used as the dye in the experiment. The primers for the target gene and reference gene are shown in Table 1.

**Table 1:** Primer sequences

Primer	Sequence	Amplification fragment size (bp)
APOA5-F	TCACCTGGGCTCTGGCTCTT	106
APOA5-R	TGCTCCACCCTGCCTTTGTC	
GAPDH-F	GGGAAACTGTGGCTTGAT	299
GAPDH-R	GAGTGGGTGTCGCTGTTGA	

### Statistical analysis

All statistical analyses in this study were conducted using R (v 4.2.1). Spearman correlation coefficient was used to assess the correlation between two factors. Additionally, LASSO regression analysis was performed with ten-fold cross-validation. A significance level of P < 0.05 was considered statistically significant.

## Results

### The screening and independence evaluation of hub genes.

**Fig. 2:**
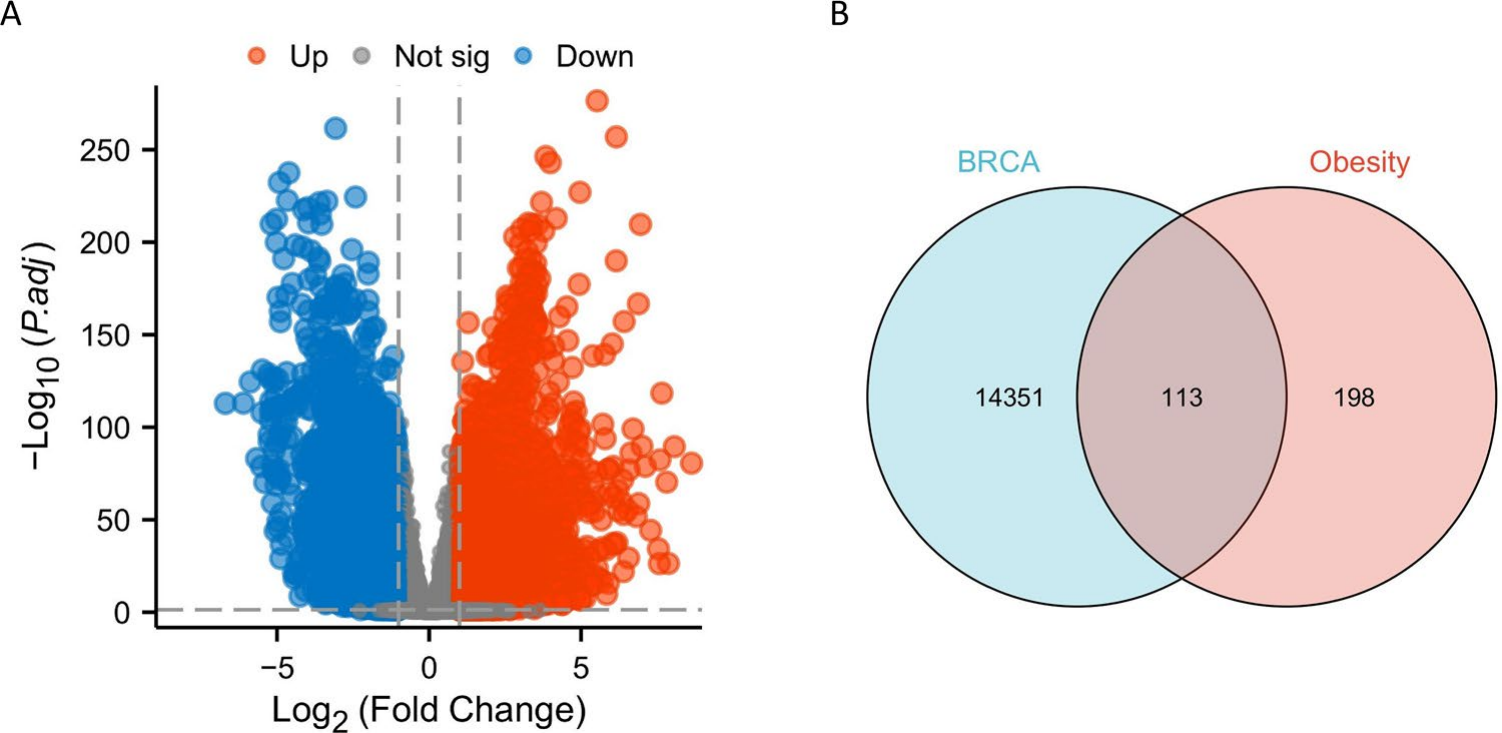
Obtain intersecting genes. **A** Volcano map. **B** Venn diagram

Firstly, differential analysis was performed on RNA-seq transcriptome data from human breast tissue samples, resulting in 14,620 differentially expressed genes. A volcano plot was generated to visualize the overall distribution of gene expression differences (Fig. 2A). The intersection of these differentially expressed genes with 311 obesity-related genes yielded 133 overlapping genes (Fig. 2B).

**Fig. 3:**
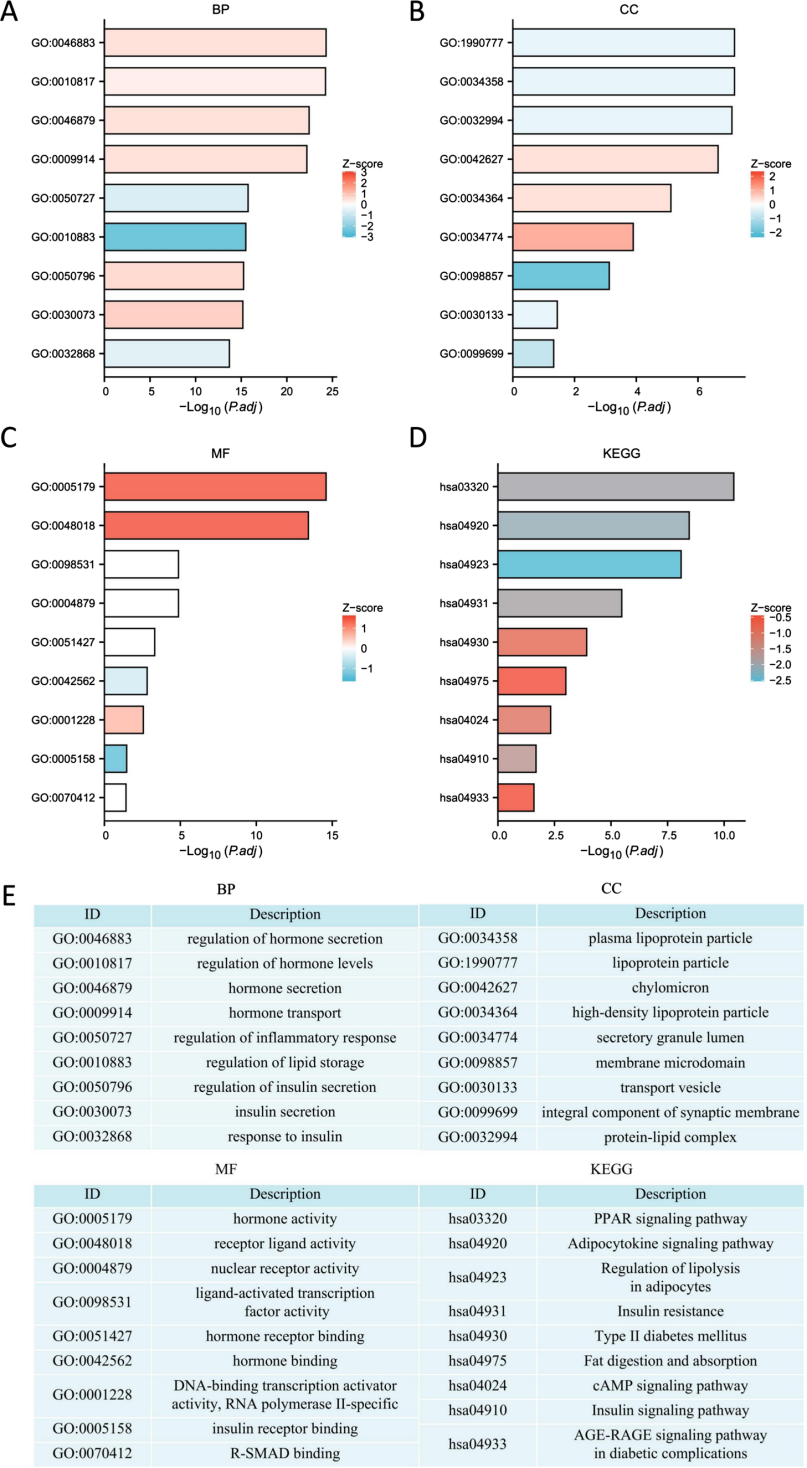
Enrichment analysis. **A** GO enrichment analysis—biological processes (BP). **B** GO enrichment analysis—cellular components (CC). **C** GO enrichment analysis—molecular functions (MF). **D** KEGG enrichment analysis. **E** Pathway ID lookup table

To investigate the main regulatory mechanisms of the 113 obesity-related genes in breast cancer patients, GO enrichment analysis and KEGG enrichment analysis were performed. In terms of biological processes (BP), the 113 genes were primarily associated with hormone secretion regulation, hormone level regulation, hormone secretion, hormone transport, regulation of inflammatory response, regulation of lipid storage, regulation of insulin secretion, insulin secretion, and response to insulin (Fig. 3A). In terms of cellular components (CC), these 113 genes were mainly associated with plasma lipoprotein particle, lipoprotein particle, chylomicron, high-density lipoprotein particle, secretory granule lumen, membrane microdomain, transport vesicle, synaptic membrane, and protein-lipid complex (Fig. 3B). In terms of molecular function (MF), the 113 genes were primarily associated with hormone activity, receptor ligand activity, nuclear receptor activity, ligand-activated transcription factor activity, hormone receptor binding, hormone binding, DNA-binding transcription activation activity, RNA polymerase II-specific activity, insulin receptor binding, and R-SMAD binding (Fig. 3C). Additionally, KEGG enrichment analysis revealed significant associations between these 113 genes and PPAR signaling pathway, adipocytokine signaling pathway, regulation of adipocyte lipolysis, insulin resistance, type II diabetes, fat digestion and absorption, cAMP signaling pathway, insulin signaling pathway, and AGE-RAGE signaling pathway in diabetic complications (Fig. 3D). The biomolecular signaling pathway ID number checklist was shown in Fig. 3E. Proteins are the products of gene expression. In order to understand the relationships between genes, a protein–protein interaction (PPI) network of 113 intersecting genes was constructed using the STRING database (Supplementary Figure 1). PPIs with a relevance score higher than 0.7 were selected to ensure the quality of the interactions and minimize false positive results. The results showed that apolipoprotein A5 (encoded by the gene APOA5) is associated with lipase in the protein interaction network.

**Fig. 4:**
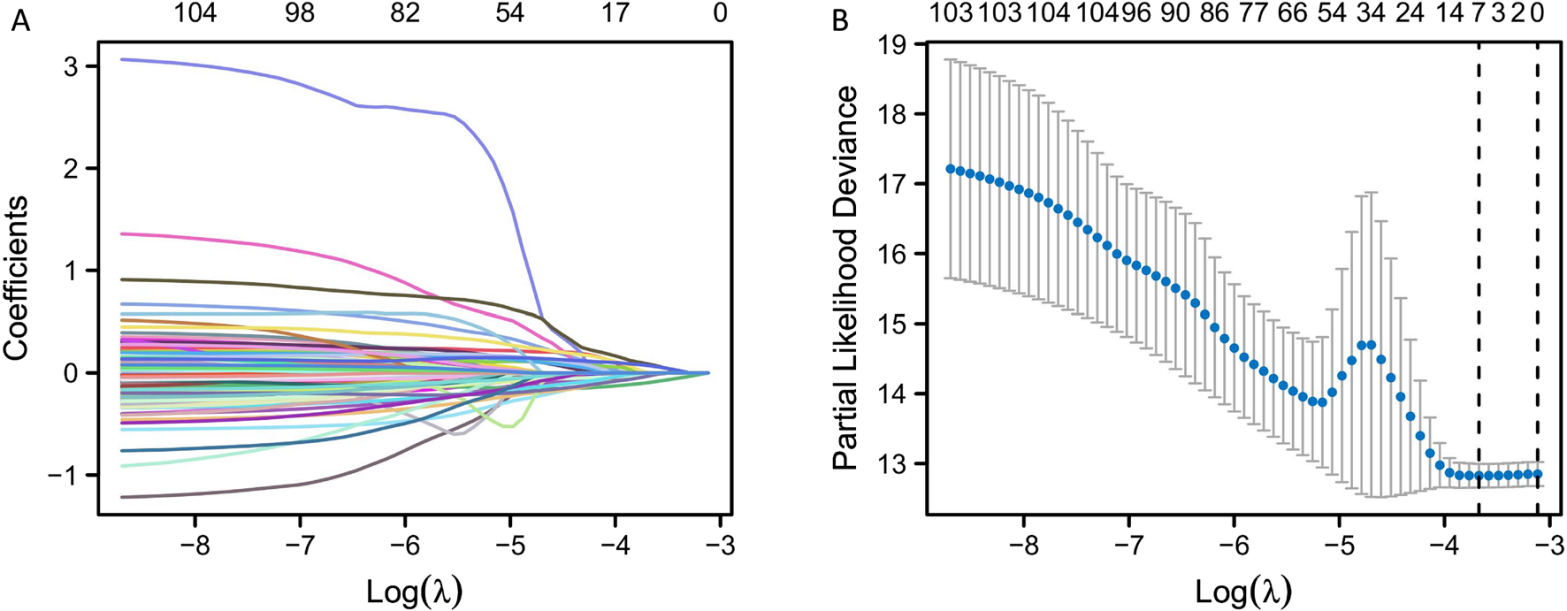
Hub gene screen. **A** LASSO variable trajectories. **B** LASSO coefficient screening

In order to further screen the 113 overlapping genes, LASSO regression analysis was performed on these genes. From the analysis results, 7 hub genes with non-zero LASSO coefficients were obtained (Fig. 4A, B).

**Fig. 5:**
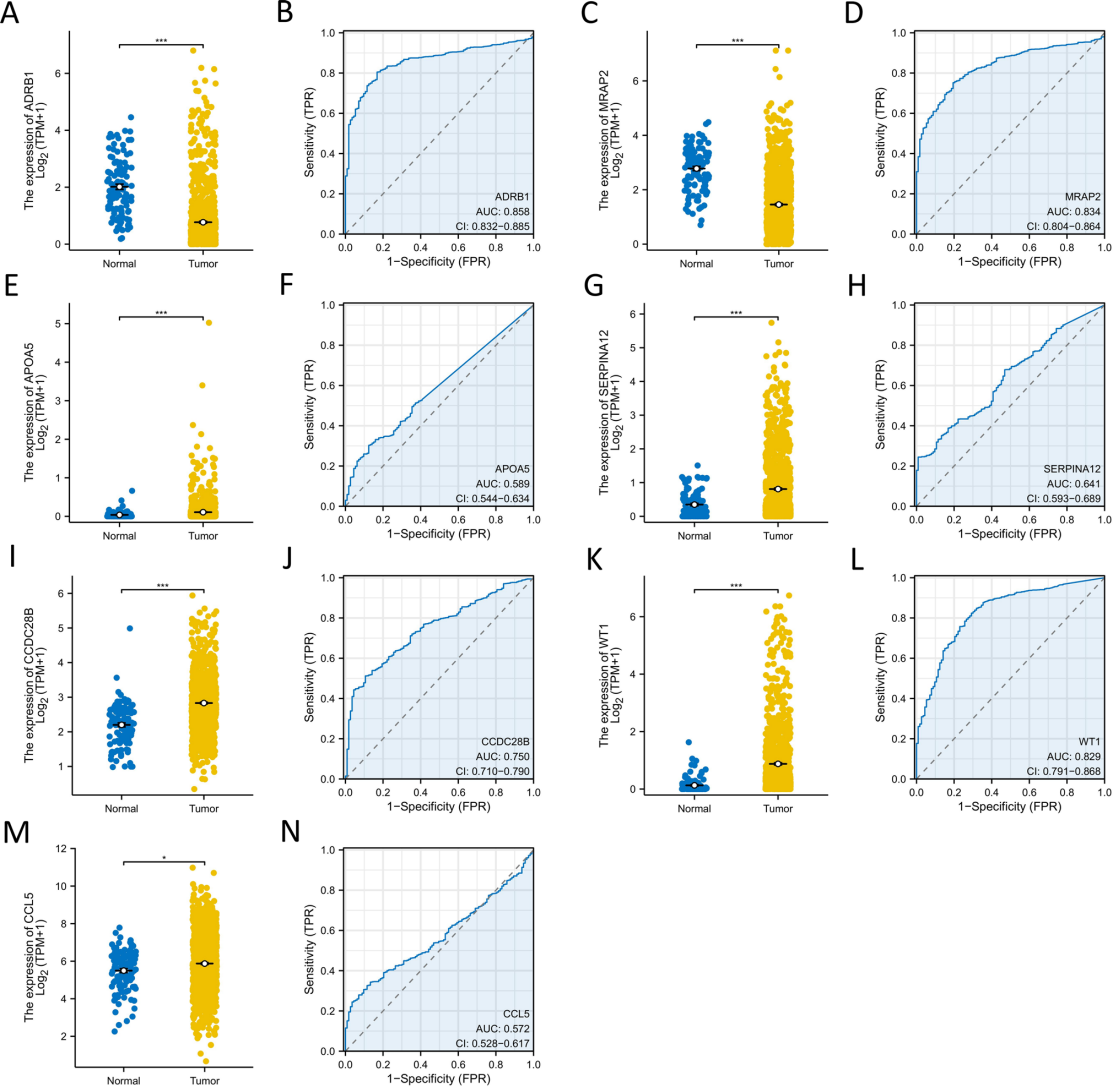
The screening of hub genes and the evaluation of their independent diagnostic value. **A** Differential analysis plot of ADRB1. **B** Diagnostic ROC curve of ADRB1. **C** Differential analysis plot of MRAP2. **D** Diagnostic ROC curve of MRAP2. **E** Differential analysis plot of APOA5. **F** Diagnostic ROC curve of APOA5. **G** Differential analysis plot of SERPINA12. **H** Diagnostic ROC curve of SERPINA12. **I** Differential analysis plot of CCDC28B. **J** Diagnostic ROC curve of CCDC28B. **K** Differential analysis plot of WT1. **L** Diagnostic ROC curve of WT1. (M) Differential analysis plot of CCL5. **N** Diagnostic ROC curve of CCL5. P < 0.05, ** P < 0.01, *** P < 0.001

To evaluate the individual effects of the 7 hub genes in BRCA patients, the expression differences of these genes in BRCA patients were analyzed (Fig. 5A–N). The results showed that 5 genes, APOA5, CCDC28B, CCL5, SERPINA12, and WT1, were significantly overexpressed in breast cancer patients (P < 0.05) (Fig. 5E, G, I, K, M). In addition, the expression of ADRB1 and MRAP2 in breast cancer patients was significantly lower than in normal samples (P < 0.05) (Fig. 5A, C). Moreover, ADRB1, CCDC28B, MRAP2, SERPINA12, and WT1 were able to effectively distinguish between normal breast tissue and cancer tissue (AUC > 0.6) (Fig. 5B, D, H, J, L).

**Fig. 6:**
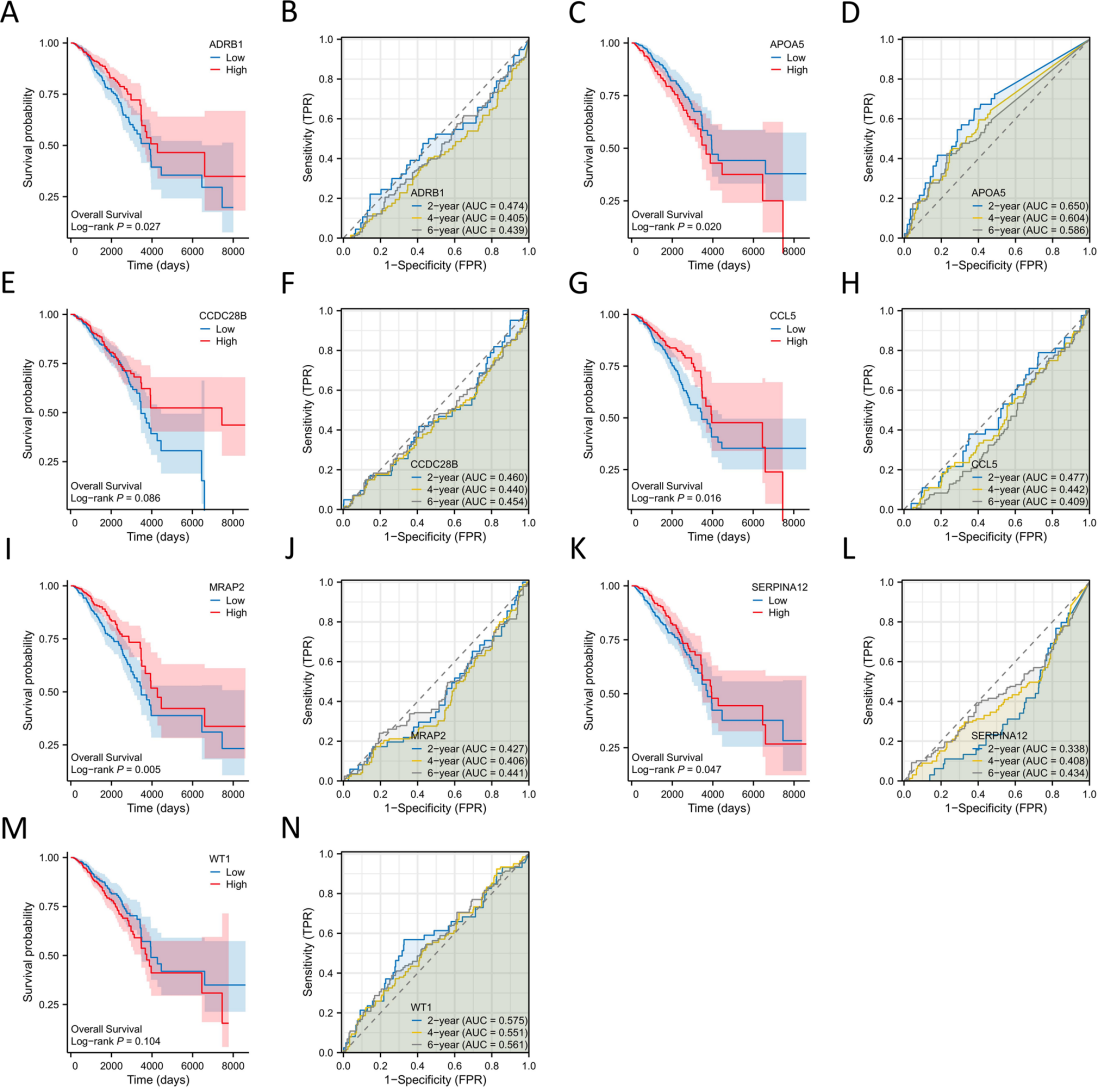
The establishment of the independent prognostic model. **A** Survival curve of ADRB1. **B** Time-dependent ROC curve of ADRB1. **C** Survival curve of APOA5. **D** Time-dependent ROC curve of APOA5. **E** Survival curve of CCDC28B. **F** Time-dependent ROC curve of CCDC28B. **G** Survival curve of CCL5. **H** Time-dependent ROC curve of CCL5. **I** Survival curve of MRAP2. **J** Time-dependent ROC curve of MRAP2. **K** Survival curve of SERPINA12. **L** Time-dependent ROC curve of SERPINA12. **M** Survival curve of WT1. **N** Time-dependent ROC curve of WT1

To further investigate the independent prognostic value of these 7 hub genes in breast cancer patients, independent survival prognostic models were constructed for each gene (Fig. 6A–N). Kaplan–Meier curves showed that only 5 genes, ADRB1, APOA5, CCL5, MRAP2, and SERPINA12, had statistically significant prognostic value (P < 0.05) in the survival analysis (Fig. 6A, C, G, I, K). Additionally, to validate the accuracy of the 7 independent survival prognostic models in predicting survival at 2, 4, and 6 years, time-dependent ROC curves were used (Fig. 6B, D, F, H, J, L, N). The results showed that the AUC values for these 7 hub genes at 2, 4, and 6 years were not high, indicating that their independent prognostic value in breast cancer patients is not significant.

### The construction of the risk prognosis model

**Fig. 7:**
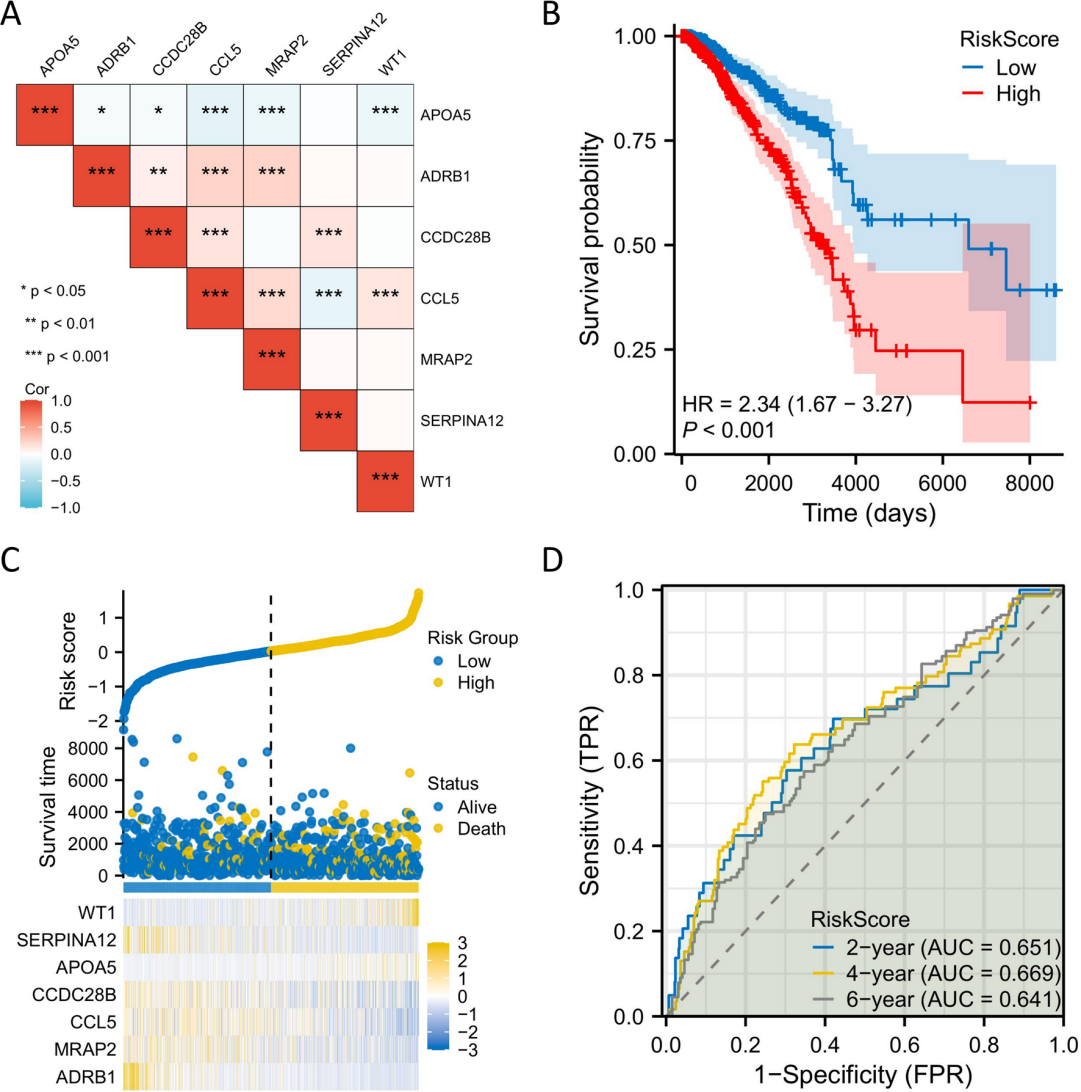
The risk prognosis model. **A** The correlation analysis of 7 hub genes. **B** Survival analysis of high-risk group and low-risk group. **C** Risk score distribution for each breast cancer patient. **D** Time-dependent ROC curves for 2, 4, and 6 years

From the above analysis, it can be concluded that the independent prognostic effects of the 7 hub genes are poor. However, through correlation analysis, we found that there were indeed potential connections among these 7 hub genes (Fig. 7A). Therefore, Cox univariate and multivariate regression analysis was conducted on the 7 hub genes (Table 2). Subsequently, the risk coefficients were calculated based on the Cox univariate and multivariate regression analysis of the 7 hub genes, and the risk prognosis model was established. Then, using the median of the risk scores obtained from the modeling as the threshold, patients were divided into high-risk and low-risk groups. Survival analysis was performed on the data of the high-risk and low-risk groups, and the Kaplan–Meier survival curve showed that the survival rate of breast cancer patients in the high-risk group was significantly lower than that in the low-risk group (P < 0.05) (Fig. 7B). This preliminary result reflects the predictive value of the model for the clinical survival period of breast cancer patients. The risk factor plot (Fig. 7C) was generated based on the modeling risk scores and survival time of each breast cancer patient when the 7 hub genes act together. Additionally, to further validate the accuracy of the prognosis model, a time-dependent ROC curve was plotted to evaluate the predictive accuracy of the model (Fig. 7D). It was found that the AUC values of the time-dependent ROC curves at 2, 4, and 6 years were all greater than 0.6, further indicating that the model can serve as a prognostic biomarker for BRCA patients when the 7 hub genes act together.

**Table 2:** The risk coefficients of the 7 hub genes

Gene symbol	Coef
ADRB1	−0.27143
MRAP2	−0.11944
CCL5	−0.12512
CCDC28B	−0.12763
APOA5	0.2303
SERPINA12	−0.26841
WT1	0.17234

### Analysis of the immune microenvironment

**Fig. 8:**
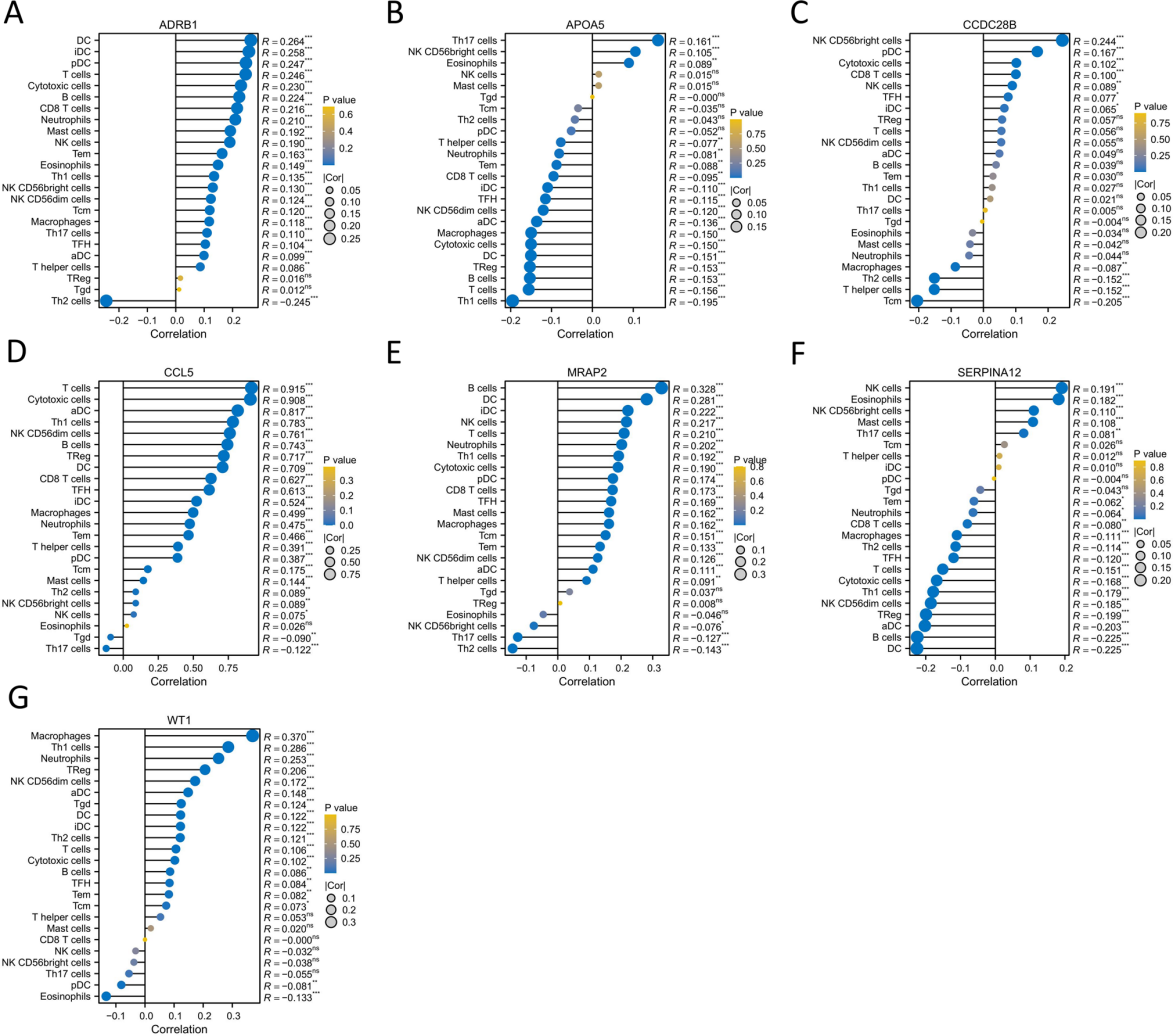
Correlation analysis of 7 HUB genes and 24 immune cells. **A–G** Correlation between 7 HUB genes and immune cells

To investigate the relationship between 7 hub genes and the immune microenvironment in BRCA, we first analyzed the correlation between the 7 hub genes and 24 types of immune cells (Fig. 8A–G). The results showed that genes ADRB1 and CCL5 were significantly positively correlated with almost all immune cells (Fig. 8A, D). Gene APOA5 was significantly negatively correlated with CD8 T cells and DC (Fig. 8B), while gene WT1 was significantly positively correlated with immune cell DC (Fig. 8G).

**Fig. 9:**
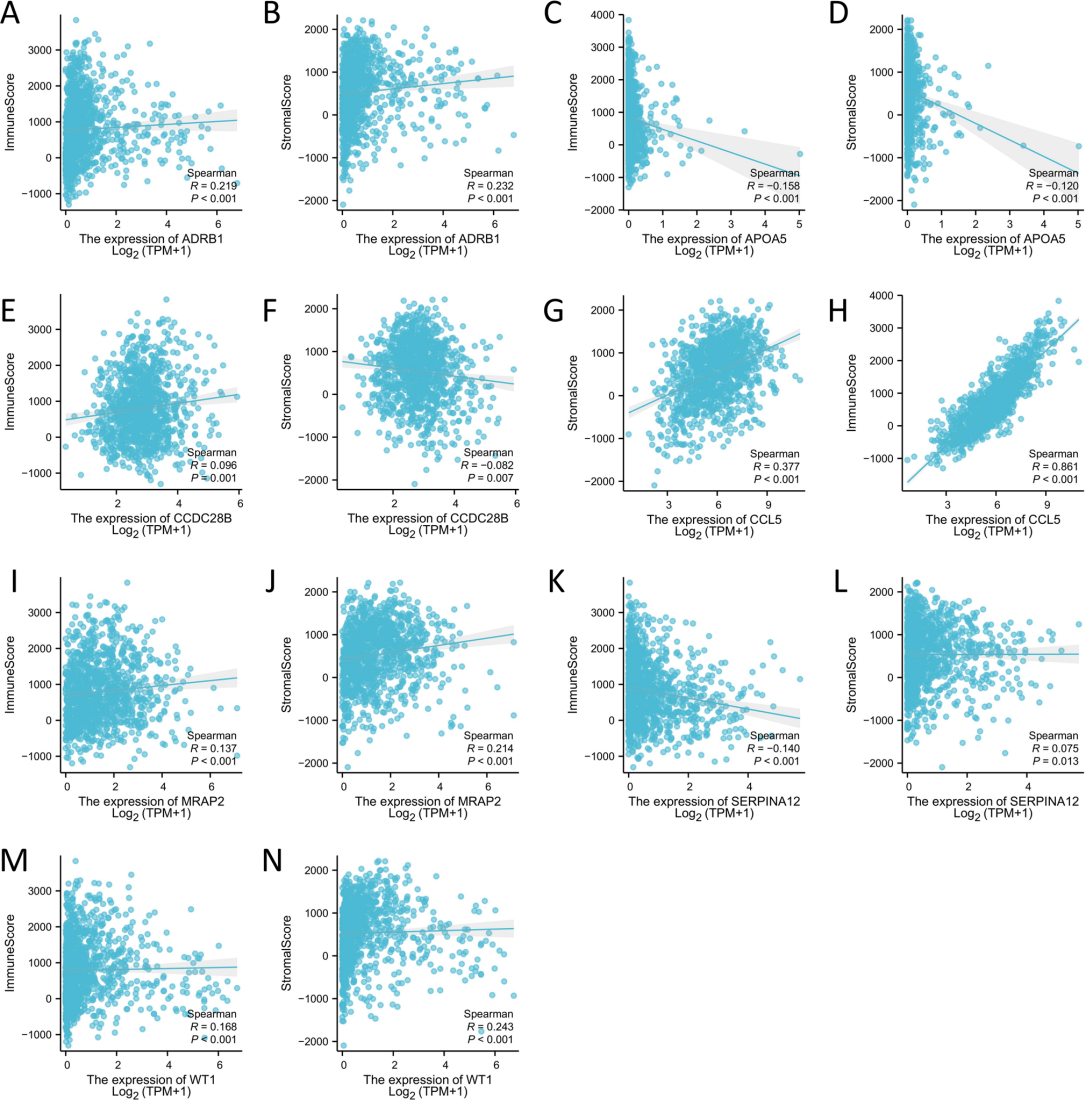
The relationship between 7 hub genes and immune score and stromal score in BRCA. **A** The correlation between ADRB1 and immune score. **B** The correlation between ADRB1 and stromal score. **C** The correlation between APOA5 and immune score. **D** The correlation between APOA5 and stromal score. **E** The correlation between CCDC28B and immune score. **F** The correlation between CCDC28B and stromal score. **G** The correlation between CCL5 and stromal score. **H** The correlation between CCL5 and immune score. **I** The correlation between MRAP2 and immune score. **J** The correlation between MRAP2 and stromal score. **K** The correlation between SERPINA12 and immune score. **L** The correlation between SERPINA12 and stromal score. **M** The correlation between WT1 and immune score. **N** The correlation between WT1 and stromal score

Next, the immune score and stromal score of the 7 hub genes were calculated based on the ESTIMATE algorithm. From the analysis results, it can be seen that 5 genes were significantly positively correlated with the immune score: ADRB1, CCDC28B, CCL5, MRAP2, and WT1 (P < 0.01, Fig. 9A, E, H, I, M), while genes APOA5 and SERPINA12 were significantly negatively correlated with the immune score (P < 0.01, Fig. 9C, K). At the same time, 5 genes were significantly positively correlated with the stromal score: ADRB1, CCL5, MRAP2, SERPINA12, and WT1 (P < 0.05, Fig. 9B, G, J, L, N), while genes APOA5 and CCDC28B were significantly negatively correlated with the stromal score (P < 0.01, Fig. 9D, F).

### The analysis of protein level

**Fig. 10:**
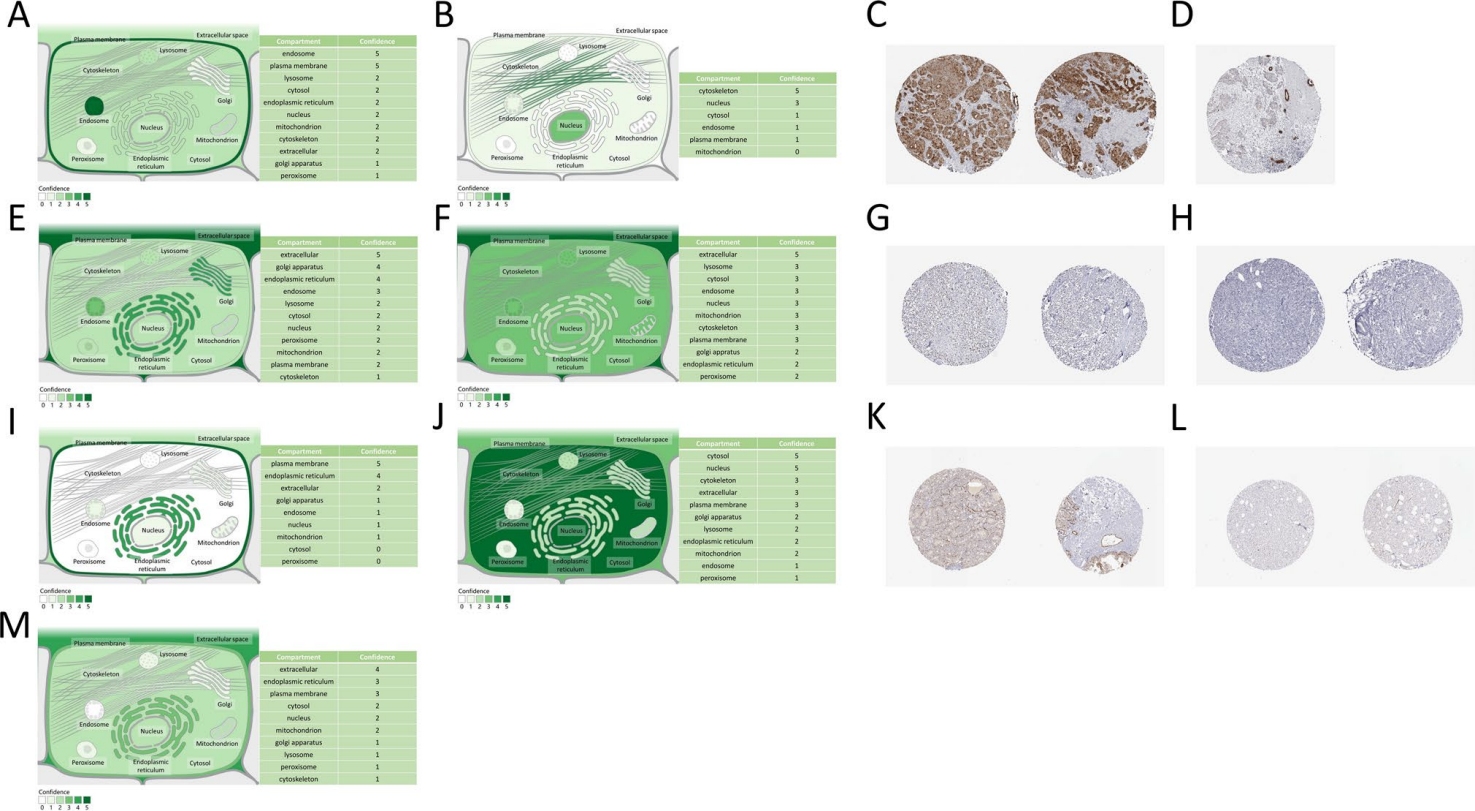
The analysis of hub gene protein expression levels. **A** Subcellular localization of ADRB1. **B** Subcellular localization of CCDC28B. **C** Strong positive staining cells for CCDC28B. **D** Negative cells for CCDC28B. **E** Subcellular localization of APOA5. **F** Subcellular localization of CCL5. **G** Cells showing moderate positive staining for CCL5. **H** Negative cells for CCL5. **I** Subcellular localization of MRAP2. **J** Subcellular localization of WT1. **K** Cells showing weak positive staining for WT1. **L** Negative cells for WT1. **M** Subcellular localization of SERPINA12

To investigate the impact of the proteins encoded by these 7 hub genes on BRCA patients, the subcellular distribution of these proteins was analyzed. The analysis revealed that the gene ADRB1 is mainly distributed in the nucleolus and plasma membrane (Fig. 10A), while the genes CCL5 and SERPINA12 are primarily located in the extracellular space (Fig. 10F, M). The gene CCDC28B is mainly found in the cell cytoskeleton (Fig. 10B), and the gene MRAP2 is predominantly located in the plasma membrane (Fig. 10I). It is worth noting that the gene APOA5 is mainly distributed in the Golgi apparatus, endoplasmic reticulum, and extracellular space, while the gene WT1 is primarily located in the cell nucleus and cytoplasm (Fig. 10E, J). Additionally, immunohistochemistry analysis was performed on the 7 hub genes. Immunohistochemistry data from the HPA database was only available for 3 genes: CCDC28B, CCL5, and WT1. Among them, CCDC28B showed strong positive and negative staining contrast in cancer cells (Fig. 10C, D), CCL5 exhibited moderate positive and negative staining contrast in cancer cells (Fig. 10G, H), and WT1 showed weak positive and negative staining contrast in cancer cells (Fig. 10K, L).

### Genetic variation analysis

**Fig. 11:**
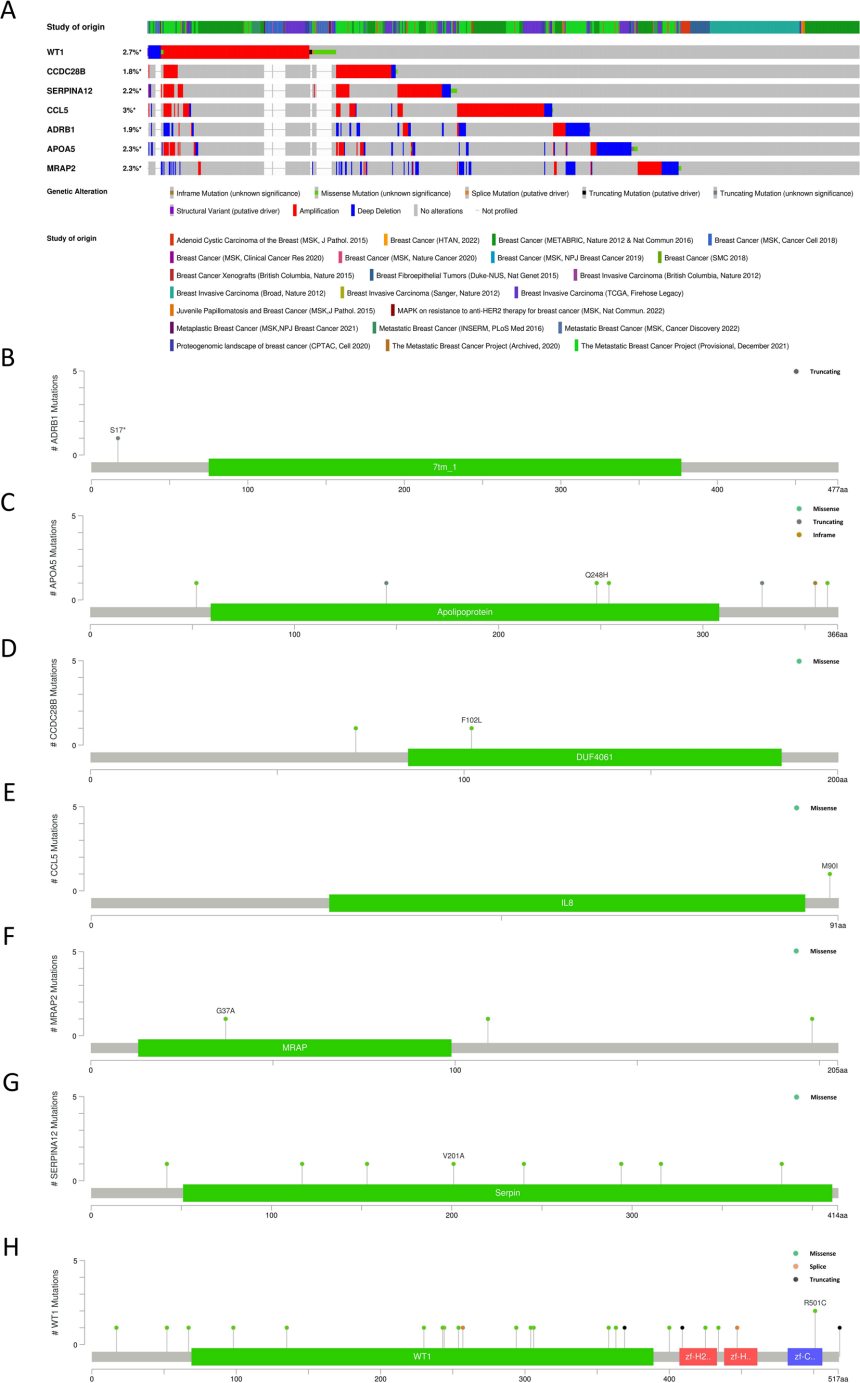
The genetic mutation analysis of hub genes. **A** Mutation data of 7 genes. **B–H** Mutation status of the protein’s two-dimensional structure corresponding to the 7 genes

According to the sample data provided by cBioPortal, genetic variation analysis was conducted on 7 hub genes. The analysis results showed that the 7 hub genes mainly exhibited amplification mutations, deep deletion mutations, and a small number of missense mutations. Among them, the amplification mutations were more prevalent in genes WT1, CCDC28B, CCL5, and SERPINA12, while the deep deletion mutations were more prominent in genes ADRB1, APOA5, and MRAP2 (Fig. 11A). Additionally, we analyzed the mutation status of the corresponding protein structures for the 7 hub genes and found that genes SERPINA12 and WT1 had a higher number of protein structure mutations. It was worth noted that no mutation sites were found in the protein structures corresponding to genes ADRB1 and CCL5, but mutations on the relevant protein structures may still affect the regulatory functions of these genes (Fig. 11B–H).

**Fig. 12:**
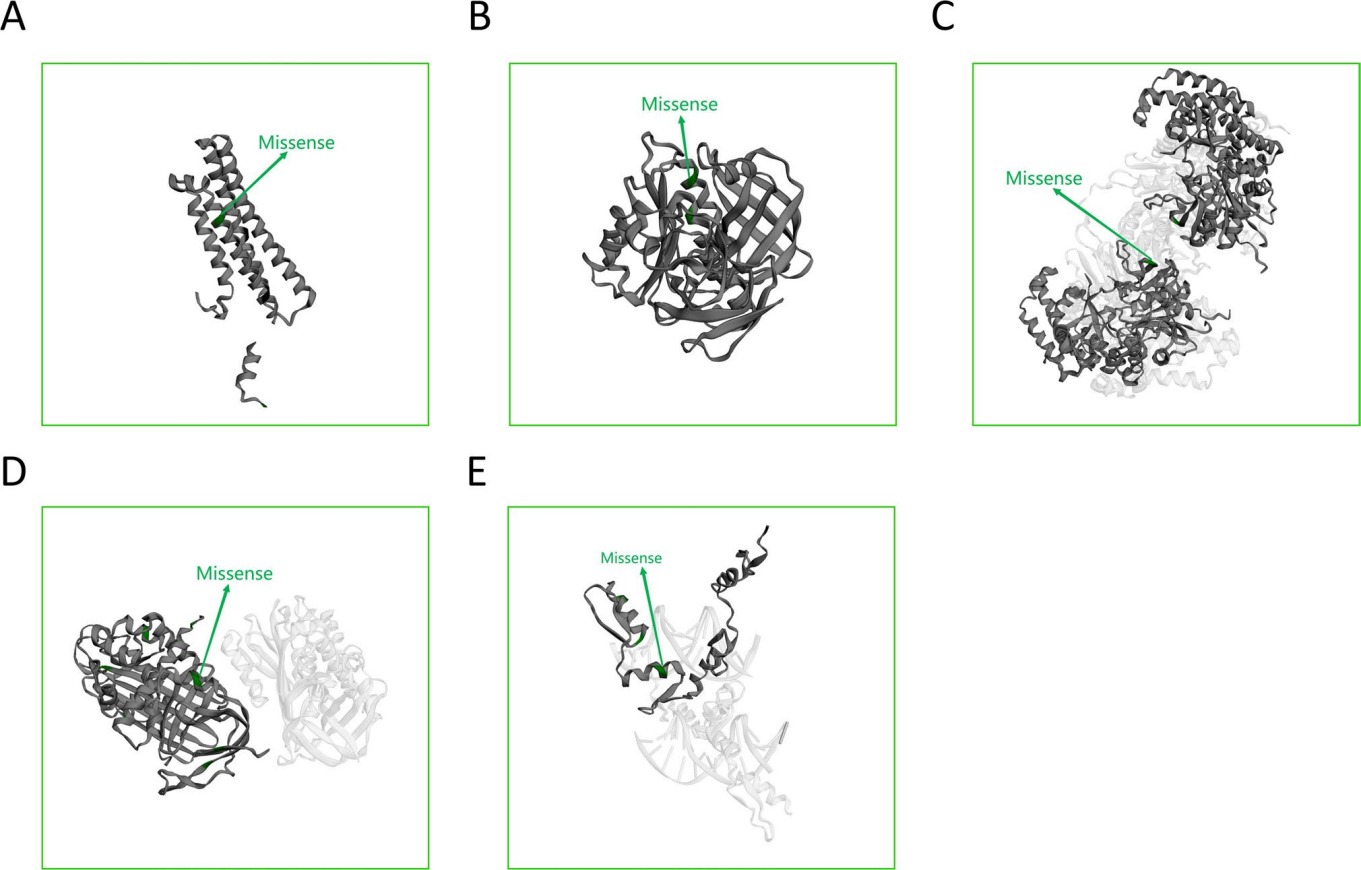
The three-dimensional structure of the protein corresponding to 7 hub genes. **A** Mutation status of the three-dimensional structure of the protein corresponding to APOA5. **B** Mutation status of the three-dimensional structure of the protein corresponding to CCDC28B. **C** Mutation status of the three-dimensional structure of the protein corresponding to MRAP2. **D** Mutation status of the three-dimensional structure of the protein corresponding to SERPINA12. **E** Mutation status of the three-dimensional structure of the protein corresponding to WT1

Furthermore, the mutation sites and types in the three-dimensional protein structures corresponding to genes APOA5, CCDC28B, MRAP2, SERPINA12, and WT1 were displayed (Fig. 12A, B, C, D, E).

### The establishment of the neural network diagnostic model

According to the risk prognosis model, the prognosis of BRCA was significantly improved when the 7 hub genes act together. Therefore, in order to investigate whether the clinical diagnostic effect of BRCA patients can also be significantly improved when the 7 hub genes act together, the neural network diagnostic model was constructed based on the RNA-seq transcriptome data of the 7 hub genes. The specific parameters of the training set and test set of the model were shown in Table 3.

**Table 3:** The parameters of the neural network diagnostic model

Setting	Accuracy	Recall	Precision	F1	AUC
Train	0.95	0.95	1.00	0.97	0.98
Value	0.94	0.97	0.97	0.97	0.94

**Fig. 13:**
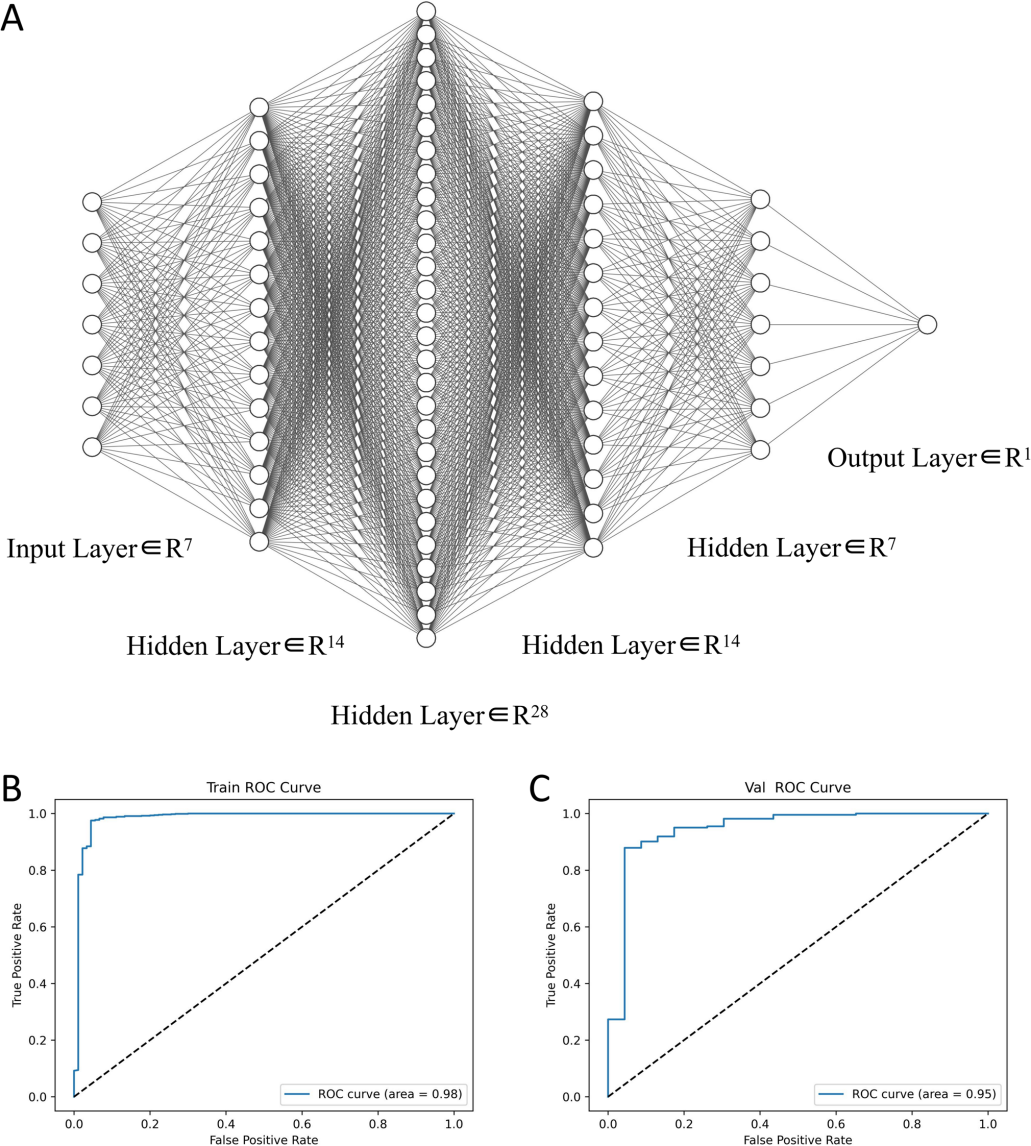
Neural network diagnostic model. **A** Neural network architecture diagram. **B** ROC curve of the training set. **C** ROC curve of the validation set

The neural network diagnostic model consists of 1 input layer with 7 neurons, 4 hidden layers with 14 neurons, 28 neurons, 14 neurons, and 7 neurons, and 1 output layer with 1 neuron (Fig. 13A). Rectified Linear Units (ReLU) were used as activation functions after each hidden layer, and the Softmax function was used as the classification function.

From the output results, it can be seen that the loss of both the training set and the test set is relatively small, and the accuracy was above 0.9, especially the AUC values were above 0.9 (Fig. 13B, C). This indicated that the diagnostic model had excellent clinical diagnostic effect for BRCA patients and can be applied to the clinical diagnosis of BRCA patients.

### The identification of BRCA subtype

**Fig. 14:**
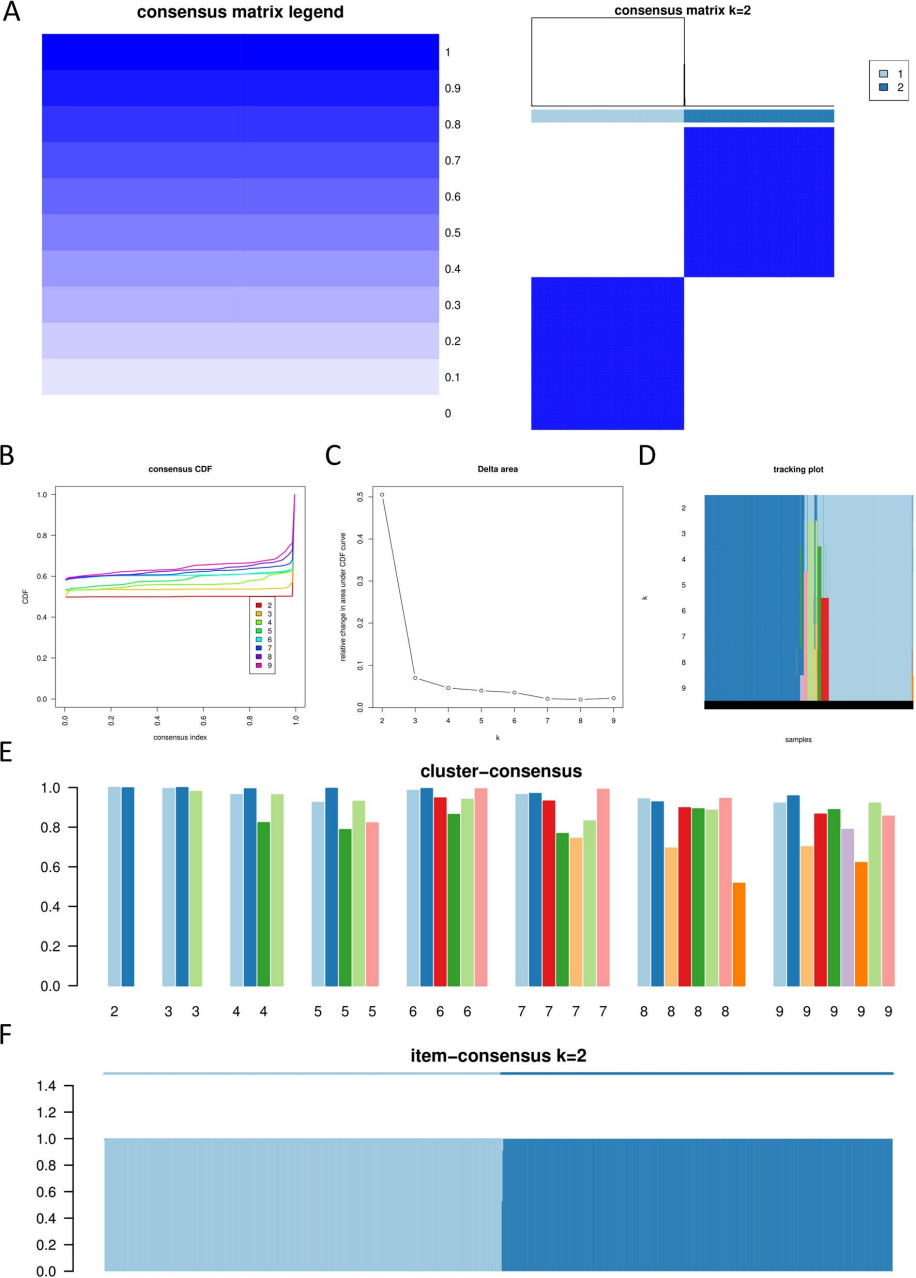
The consensus algorithm was used to identify subtypes. **A** Consensus matrix heat map. **B** Consensus cumulative distribution function (CDF). **C** Delta area. **D** Tracking plot. **E** Cluster-consensus. **F** Item-consensus—k = 2

To provide targeted treatment options for BRCA patients in clinical practice, BRCA subtypes were identified based on RNA transcriptomic data of 7 hub genes. Firstly, the sample data was subjected to clustering analysis using a consensus clustering algorithm. Then, the optimal value of k was determined as 2 using the PAC algorithm, dividing BRCA into two subtypes. Additionally, the optimal value of k as 2 was confirmed through the consensus matrix, consensus CDF, Delta area, and clustering results for different k values (Fig. 14A–C, E). Moreover, the consensus matrix, tracking plot, and item-consensus at k = 2 indicated that the two BRCA subtypes exhibited high stability and specificity (Fig. 14A, D, F).

**Fig. 15:**
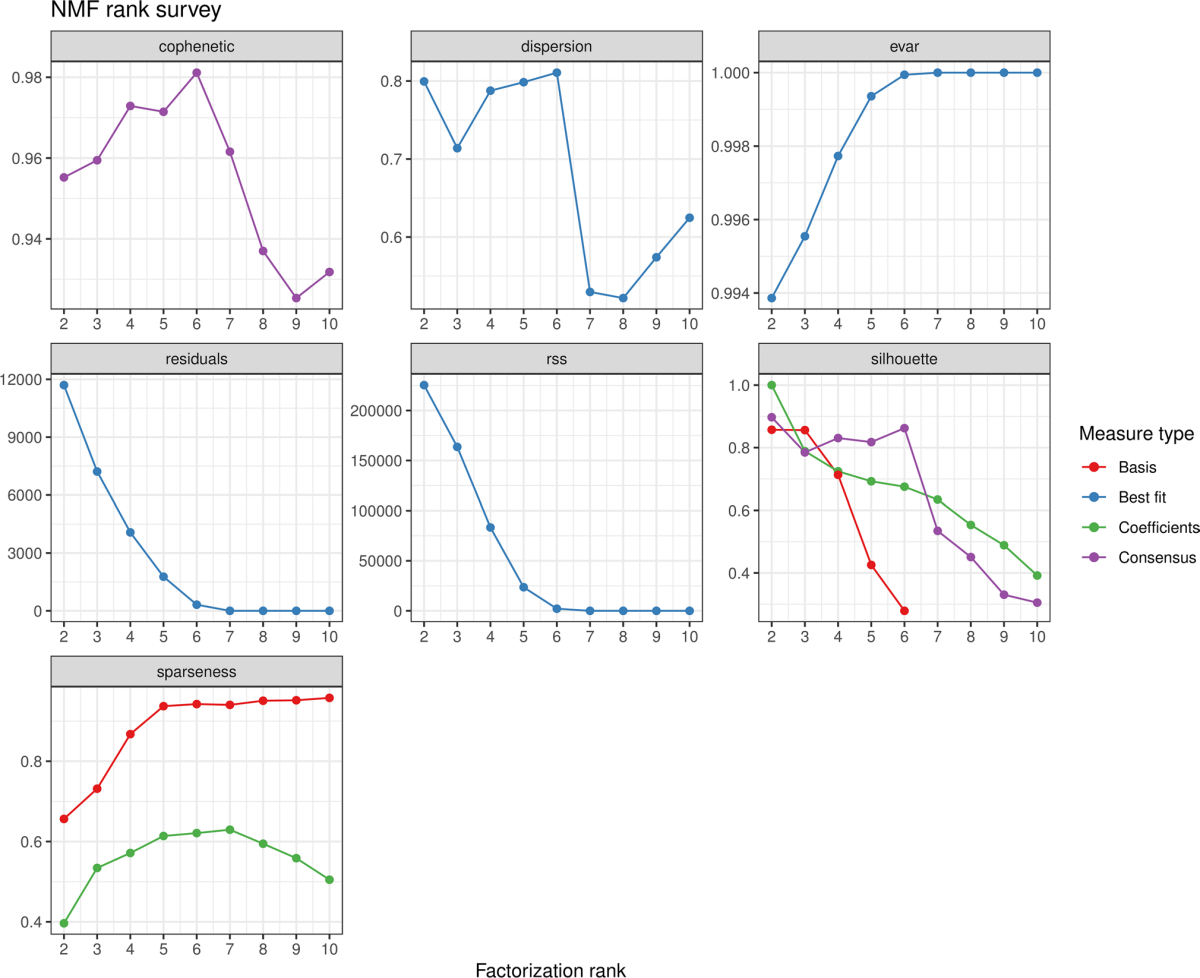
NMF rank measure plot

**Fig. 16:**
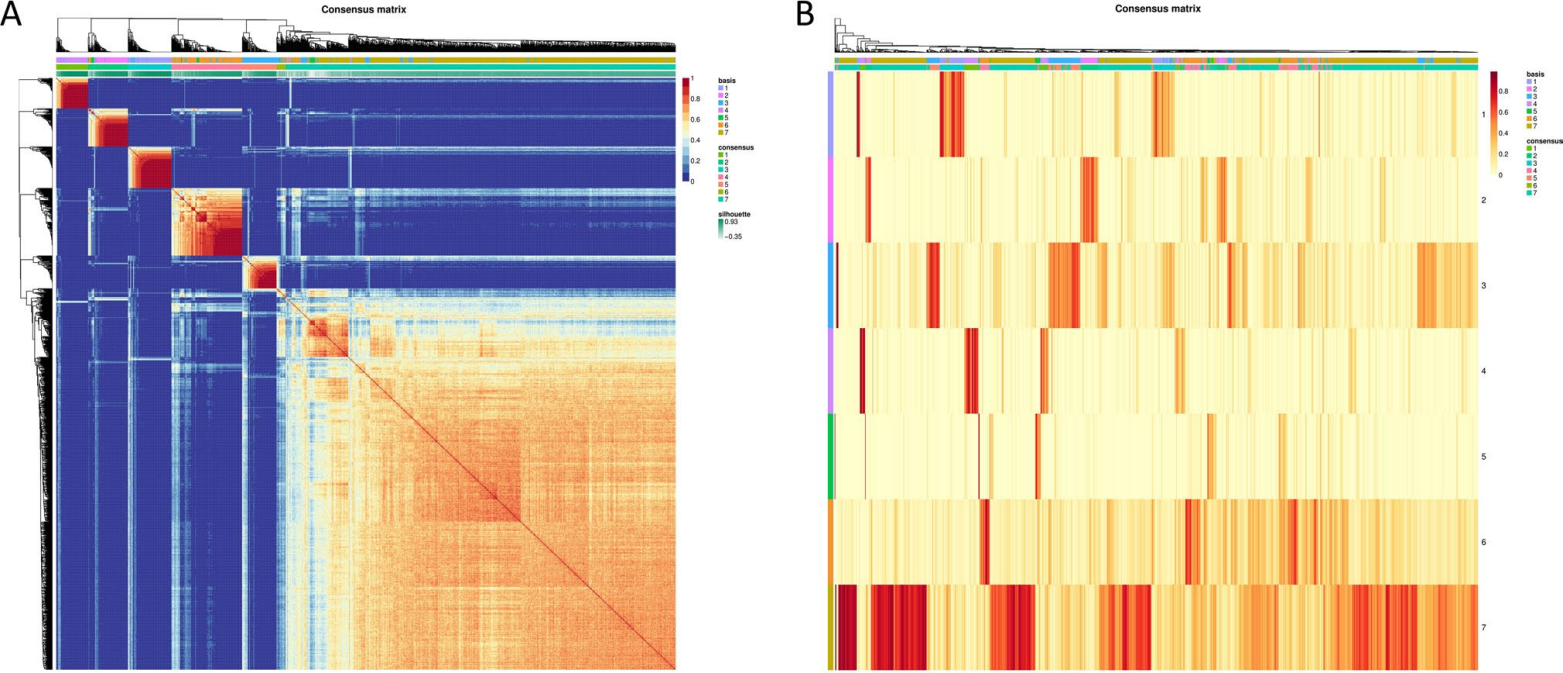
Identification of BRCA subtypes using NMF algorithm. **A** Mixing coefficient matrix. **B** Consistency matrix

**Fig. 17:**
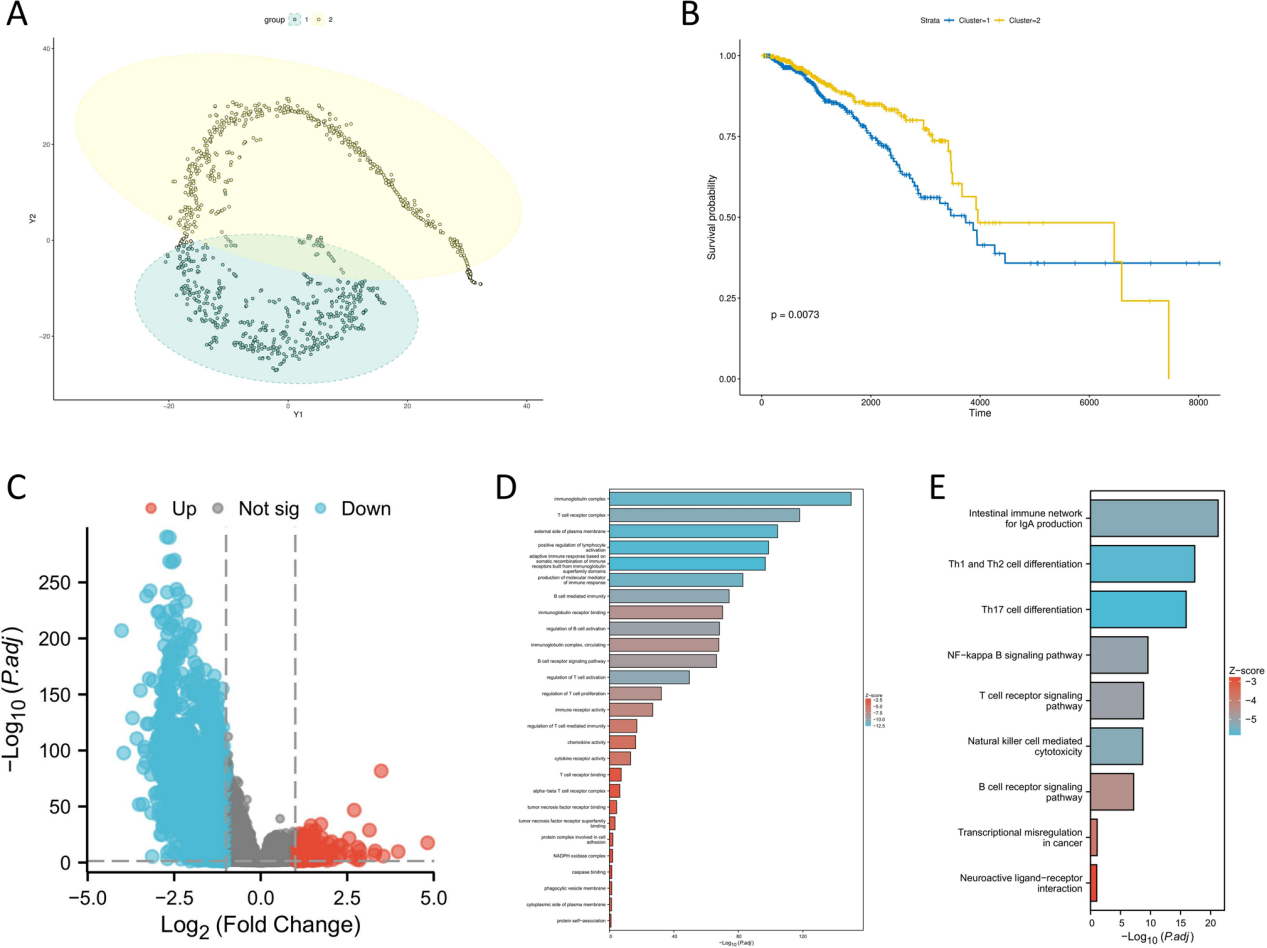
In-depth analysis of the two subtypes of BRCA. **A** t-SNE scatter plot. **B** Kaplan–Meier curve. **C** Differential analysis of the two subtypes of BRCA. **D** GO enrichment analysis—1533 differentially expressed genes. **E** KEGG enrichment analysis—1533 differentially expressed genes

Through the consensus clustering algorithm, the BRCA subtypes were initially determined, but the results obtained from a single perspective may have some randomness. Therefore, we also used non-negative matrix factorization (NMF) algorithm for subtype identification of BRCA samples. The optimal NMF rank parameter was selected based on the NMF rank measure plot and cophenetic index, with a rank value of 7 (Fig. 15). Based on the optimal rank value, the coefficient matrix plot and consensus matrix plot were generated (Fig. 16A, B). The results showed that the clustering stability was poor when the rank value was set to 7. After comparative analysis, we adopted the consensus clustering algorithm to divide breast cancer into two subtypes.

t-SNE algorithm was used to reduce the dimensionality of the high-dimensional distribution and the t-SNE scatter plot was generated. The distribution results indicated that these two subtypes clustered stably and had strong specificity (Fig. 17A). Additionally, survival analysis was conducted on these two subtypes of BRCA patients, and the results showed that the survival rate of subtype 1 patients was significantly higher than that of subtype 2 patients (Fig. 17B). Patients with both subtypes of BRCA were extracted for differential analysis based on RNA transcriptome data, resulting in 1533 differentially expressed genes (Fig. 17C). GO enrichment analysis and KEGG enrichment analysis were performed on these differentially expressed genes. The results indicate that these 1533 differentially expressed genes are mainly associated with the immune microenvironment of BRCA patients (Fig. 17D, E).

**Fig. 18:**
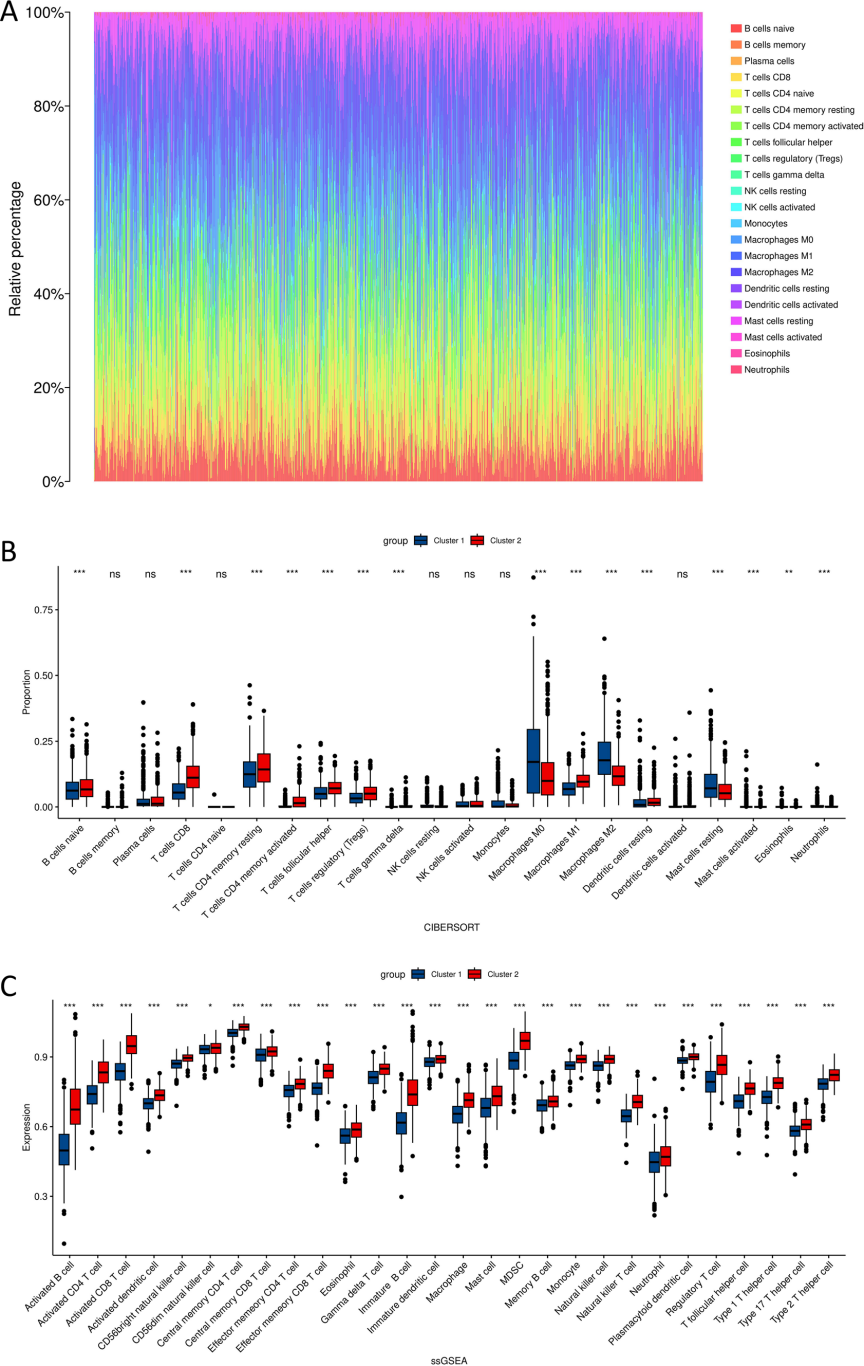
The relationship between the two subtypes of BRCA and immune cells. **A** Relative abundance plot of immune cells. **B** Differential plot of immune cell proportions between the two subtypes of BRCA. **C** Expression plot of immune cells in the two subtypes of BRCA

Therefore, it is speculated that the differences between these two subtypes can be explored from the perspective of the immune microenvironment, the CIBERSORT algorithm was used to calculate the relative abundance of 22 immune cells in each subtype of BRCA, and the results were visualized in an immune infiltration relative abundance plot (Fig. 18A). In addition, the proportions and gene expression levels of the 22 immune cells in the two BRCA subtypes were analyzed separately using the CIBERSORT algorithm and ssGSEA analysis (Fig. 18B, C). Among them, T cells had the highest proportion among the immune cells. The results showed significant differences in the proportions of immune cells between the two clusters, with a significantly higher proportion of CD8 T cells in subtype 2 compared to subtype 1. Moreover, in the immune cell expression plots of the two clusters, the expression levels of most immune cells were significantly higher in subtype 2 compared to subtype 1.

**Fig. 19:**
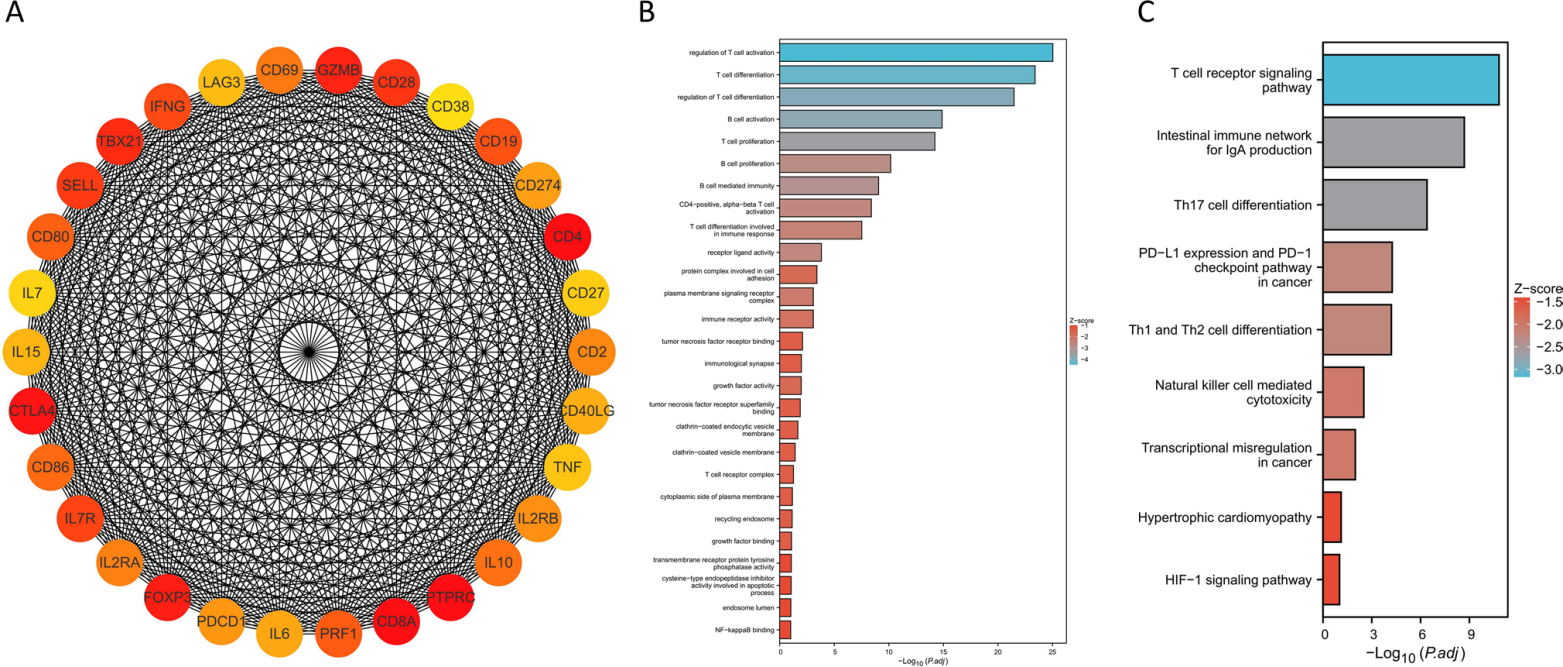
Mechanistic analysis of the 30 hub genes. **A** The PPI—30 hub genes. **B** GO enrichment—30 hub genes. **C** KEGG enrichment—30 hub genes

**Fig. 20:**
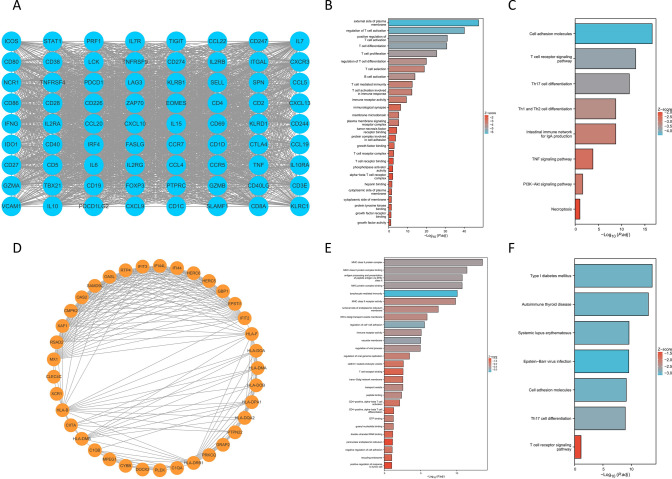
Mechanistic analysis of two subnetworks. **A** The PPI—subnetwork 1. **B** GO enrichment—subnetwork 1. **C** KEGG enrichment—subnetwork 1. **D** The PPI—subnetwork 2. **E** GO enrichment—subnetwork 2. **F** KEGG enrichment—subnetwork 2

In order to further investigate the differences in the underlying mechanisms of the two BRCA subtypes in patients, 30 hub genes were selected from the 1533 differentially expressed genes using the cytoHubba plugin in Cytoscape (Fig. 19A). Additionally, two subnetworks were identified using the MCODE plugin (Fig. 20A, D). Subnetwork 1 consists of 72 nodes and 2054 edges (Fig. 20A), while subnetwork 2 consists of 37 nodes and 211 edges (Fig. 20D). GO enrichment analysis and KEGG enrichment analysis were performed on the aforementioned genes in each subnetwork. The enrichment analysis of the 30 hub genes revealed that they were primarily associated with the regulation of the immune microenvironment in BRCA patients (Fig. 19B, C). The enriched physiological activities were mostly significantly related to immune cells. Enrichment analysis of the two subnetworks reveals some differences in their regulatory roles in BRCA patients. Subnetwork 1 tends to be involved in the regulation of B cells and T cells (Fig. 20B, C), while subnetwork 2 is more associated with intercellular material transport and regulation of lymphocytes (Fig. 20E, F).

### The analysis of single cell sequencing

**Fig. 21:**
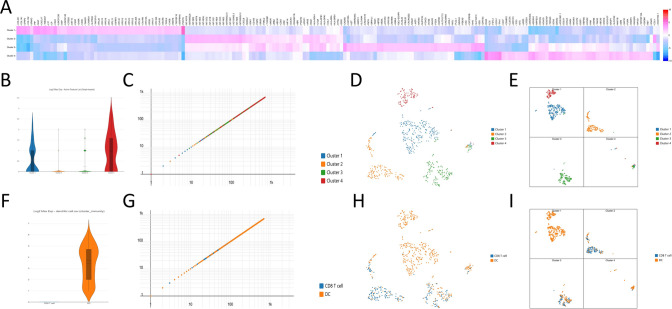
The analysis of single cell transcriptome. **A** Correlation heatmap between gene expression levels and four cell subgroups. **B** Differential analysis of four cell subgroups. **C** Feature plots of four cell subgroups. **D** t-SNE scatter plot of four cell subgroups. **E** t-SNE scatter plot of four cell subgroups (k = 4). **F** Differential analysis of two immune cell clusters. **G** Feature plots of two immune cell clusters. **H** t-SNE scatter plot based on two immune cell clusters. **I** t-SNE scatter plot based on two immune cell clusters (k = 4)

In order to study the pathogenesis of breast cancer at the single-cell level, single-cell sequencing was performed on gene data obtained from 10X Genomics. The data was divided into four cell subgroups, totaling 699 cells. The proportions of the four subgroups are as follows: Cluster 1 with 259 cells (37%), Cluster 2 with 176 cells (25%), Cluster 3 with 166 cells (24%), and Cluster 4 with 98 cells (14%). Differential analysis was conducted on these four cell subgroups, and a two-dimensional feature plot (Fig. 21B, C) was generated. The t-SNE algorithm was used for dimensionality reduction and visualization of the distribution of the four cell subgroups (Fig. 21D). Additionally, a t-SNE scatter plot was generated with k value set to 4 (Fig. 21E). Furthermore, the expression level distribution of genes in the four cell subgroups was analyzed and a heatmap was generated (Fig. 21A). Subsequently, marker genes for CD8 T cells and DC immune cells were obtained from the cell marker gene database. The four cell subgroups were re-clustered, revealing 218 CD8 T cells (31%) and 481 DC cells (69%). Differential analysis was conducted on these two cell groups, and a two-dimensional feature plot was generated (Fig. 21F, G). The high-dimensional data obtained from single-cell sequencing was reduced using the t-SNE algorithm and visualized in a t-SNE scatter plot (Fig. 21H). Additionally, a t-SNE scatter plot was generated with k value set to 4 for clearer observation of cluster boundaries (Fig. 21I).

### Real-time quantitative PCR (qRT-PCR)

**Fig. 22:**
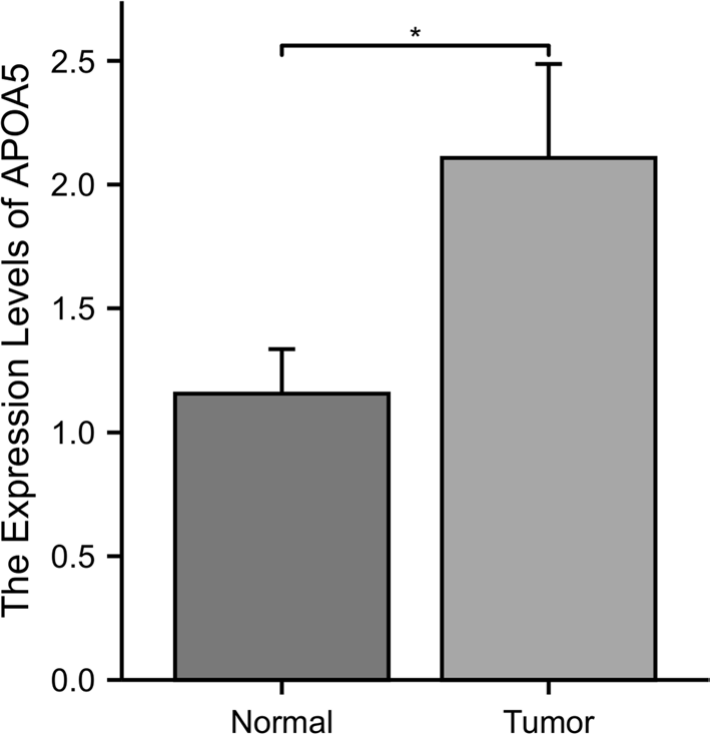
Validation of qRT-PCR experiments

By performing qRT-PCR experiments on blood samples from 11 BRCA patients and 10 healthy individuals, it was found that APOA5 was significantly upregulated in BRCA patients (Fig. 22). This result was consistent with the previous bioinformatics analysis in this study, thus providing experimental evidence to support the conclusions of this study.

## Discussion

Breast cancer was a disease with significant heterogeneity, and its biological mechanisms were not fully understood [26]. In the state of obesity, the number and size of adipocytes increase, leading to an increase in adipose tissue [27]. Additionally, excessive fat accumulation can cause dysfunction of adipose tissue, affecting the secretion of cytokines, adipokines, and hormones [28]. These changes trigger a series of biological effects, such as altered adipose tissue function, adipocyte apoptosis, chronic inflammation, and changes in the microenvironment. There was evidence from multiple previous studies confirming the association between these changes and the occurrence and development of breast cancer [29–31].

This study focuses on exploring obesity as an important risk factor for breast cancer and its role in cancer occurrence and development. It comprehensively and in-depth investigates 7 key genes shared by obesity and breast cancer, evaluating their independent diagnostic value and independent prognostic value. The risk prognosis model was established for the combined action of these 7 genes, indicating that they provided a better prognostic effect when acting together. Furthermore, the study assessed the role of these 7 genes in the immune microenvironment of BRCA patients through relevant analysis of the immune microenvironment. Through a deeper investigation of these 7 key genes, GO enrichment analysis was conducted from biological processes, cellular levels, and molecular functions, as well as KEGG enrichment analysis, to explore the biological significance of these genes in breast cancer. GO enrichment analysis revealed the diverse roles of these genes in normal cell physiology and tumor development, including cell proliferation, cell cycle regulation, and signal transduction. KEGG enrichment analysis provided a more macroscopic perspective, revealing the roles of these genes in key signaling pathways of breast cancer, such as the PPAR signaling pathway, adipocytokine signaling pathway, regulation of adipocyte lipolysis, insulin resistance, type II diabetes, fat digestion and absorption, cAMP signaling pathway, insulin signaling pathway, and AGE-RAGE signaling pathway in diabetes complications. Adipocytokines (also known as adipokines), such as leptin and adiponectin, can induce inflammation in the body [32, 33]. Previous studies have shown that peroxisome proliferator-activated receptors (PPARs) have regulatory effects on inflammation, inhibiting the expression of various inflammatory mediators, regulating the activation state of inflammatory cells, and affecting the differentiation and function of inflammatory cells [34, 35]. Additionally, in a high-glucose environment, the binding of advanced glycation end products (AGEs), the final products of non-enzymatic glycation reactions, to their receptor RAGE can activate intracellular inflammatory signaling pathways, promote inflammatory reactions, and exacerbate the progression of chronic diseases such as diabetes [36, 37]. Based on these findings, it can be inferred that obesity may increase the risk of breast cancer by triggering inflammation.

In order to provide precise treatment for BRCA patients in clinical practice, based on the RNA transcriptome data of 7 key genes, 1226 BRCA patients were divided into two BRCA subtypes. By combining survival data, it was found that patients in subtype 1 had a poor prognosis. Furthermore, to further explore the differences between the two subtypes, differential analysis was performed based on the RNA transcriptome data, resulting in 1533 differentially expressed genes. Through the study of 30 hub genes and the mechanisms of two subnetworks among the 1533 differentially expressed genes, it was discovered that the two BRCA subtypes mainly differ in the regulatory mechanisms within the immune microenvironment of BRCA patients. Subtype 1 was mainly associated with the physiological activities of B cells and T cells, while subtype 2 was associated with intercellular interactions and lymphocytes. This provided new perspectives and possibilities for improving the understanding and treatment strategies for BRCA in the future. However, further research was needed to fully understand the differences in the pathogenesis between the two subtypes.

It is worth noting that these seven hub genes have received strong support from previous studies. APOA5 has been confirmed to be a novel regulator of lipid storage in adipocytes, playing a crucial role in lipid metabolism and systemic energy homeostasis [38]. Moreover, alterations in APOA5 can elevate the risk of developing breast cancer [39]. The transcription factor WT1 had also attracted attention, as its target genes include growth factors and cell division regulatory factors. Interestingly, it contained an inhibitory domain that can suppress the function of other transcriptional activators [40–42]. Similarly, we also observed immune genes ADRB1 and chemokine ligand CCL5, which were associated with breast cancer prognosis. ADRB1 was considered an important target in various therapeutic applications, and studies have shown that its overexpression in breast cancer tissue may enhance tumor sensitivity to β-blockers and improve prognosis [43, 44]. CCL5 was highly expressed in white adipose tissue and may promote breast cancer development through inflammation and immune pathways [45–47]. In the study of obesity and insulin sensitivity, mutations in SERPINA12 and MRAP2 have caught our attention. The expression of SERPINA12 was positively correlated with obesity and insulin sensitivity, while mutations in MRAP2 were significantly associated with increased obesity risk [48, 49]. It was worth mentioned that we also discovered the gene CCDC28B, which may play an important role in gastric cancer development [50]. However, to date, there have been no reports on its potential role in breast cancer.

Through the above analysis, we have found that obesity was indeed a risk factor for BRCA patients and have identified 7 new biomarkers for diagnosis. However, we must acknowledge certain limitations of this study. Firstly, the biological mechanisms of breast cancer are still complex and not fully understood, which made predictions and interventions based on these mechanisms challenging. While our study provides new perspectives and potential clues for the diagnosis, treatment, and prognosis assessment of breast cancer in the future, these findings require further clinical validation and in-depth research. Additionally, although we have identified some important breast cancer-related genes, their specific roles in disease development and how they impacted breast cancer treatment and prognosis still need further exploration. For example, the role of the gene CCDC28B has been confirmed in gastric cancer, but its role in breast cancer has not been discovered. Overall, while our study provides valuable findings and insights, it was important to recognize the limitations of these findings and conduct further in-depth and expanded research in the future to provide more comprehensive and accurated personalized treatment strategies for breast cancer patients.

## Conclusion

Breast cancer was a complex and significantly heterogeneous disease, with many mysteries surrounding its etiology and progression waiting to be unraveled. This study focuses on obesity, a major risk factor that has a significant impact on breast cancer, and delves into key genes, molecular subtypes, and immune cell infiltration characteristics of breast cancer. The aim was to reveal new areas of disease understanding from this novel perspective and provide possible directions for optimizing treatment strategies.

Our findings revealed that obesity can trigger an increase in the number and size of adipocytes, leading to a series of biological and inflammatory reactions that ultimately shape an environment favorable for cancer development. Through single and multiple factor Cox analysis and LASSO coefficient screening of obesity-related BRCA differential genes, we successfully identified 7 key genes that play important roles in the occurrence and progression of breast cancer. These genes had the potential to become new therapeutic targets. Furthermore, through unsupervised clustering analysis, we revealed the existence of two major molecular subtypes of breast cancer, which showed significant differences in the survival prognosis of BRCA patients. The two BRCA subtypes may also have differential regulation in the immune microenvironment of BRCA patients. This result suggested that these two subtypes may have different biological characteristics and clinical manifestations, providing a theoretical basis for achieving precision treatment of BRCA.

However, we must recognize that this study has some limitations. Although this study presented some promising new findings that had the potential to change the future diagnosis, treatment, and prognosis assessment of breast cancer, these research findings still require further clinical validation and in-depth exploration. It was hoped that through future research, we can provide more comprehensive and accurate personalized treatment plans for breast cancer patients.

### Supplementary Information

Supplementary file1 (DOCX 1232 KB)

## Data Availability

The data used to support this study is available from the corresponding author upon request.
