# Supplementary figures and images for: Stratification and prognostic evaluation of breast cancer subtypes defined by obesity-associated genes
Source: Discov Oncol. 2024 Apr 27;15:133. doi: 10.1007/s12672-024-00988-0 (PMC11055831; doi:10.1007/s12672-024-00988-0)

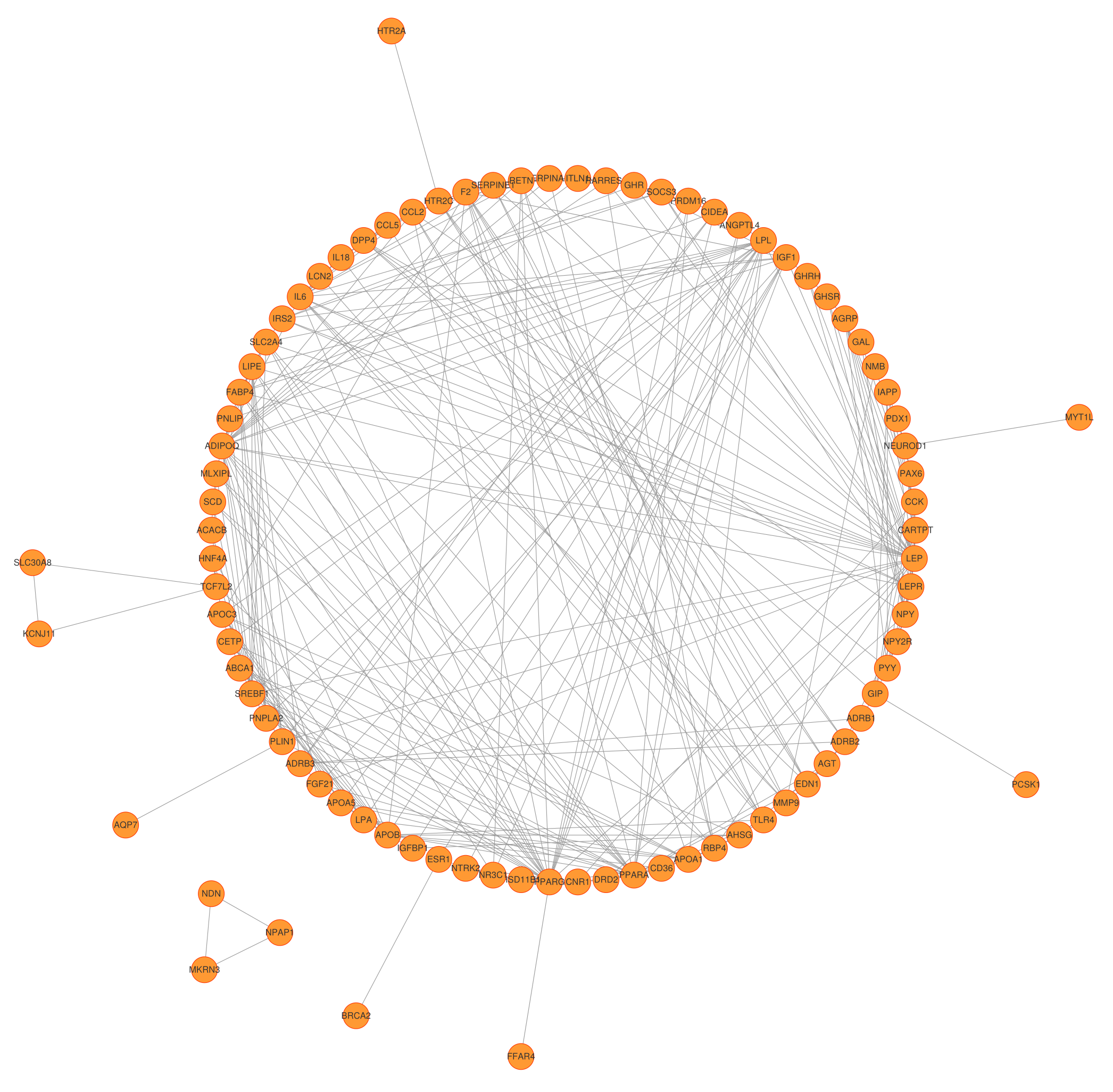


**Supplemental Figure 1 |** The PPI of 113 intersecting genes.

Supplement: Supplementary file 1 — Supplementary file1 (DOCX 1232 KB) [file 12672_2024_988_MOESM1_ESM.docx]
